# Heteroepitaxial MOF-on-MOF Photocatalyst for Solar-Driven Water Splitting

**DOI:** 10.1021/acsnano.4c03442

**Published:** 2024-07-30

**Authors:** Thibaut Le Huec, Antón López-Francés, Isabel Abánades Lázaro, Sergio Navalón, Herme G. Baldoví, Mónica Giménez-Marqués

**Affiliations:** † Instituto de Ciencia Molecular (ICMol), Universidad de Valencia, C/Catedrático José Beltrán Martínez, 2, 46980 Paterna, Valencia, Spain; ‡ Departamento de Química, Universitat Politècnica de València, C/Camino de Vera, s/n, 46022 Valencia, Spain

**Keywords:** MOF-on-MOF, heterostructure, photocatalyst, overall water splitting, sunlight irradiation

## Abstract

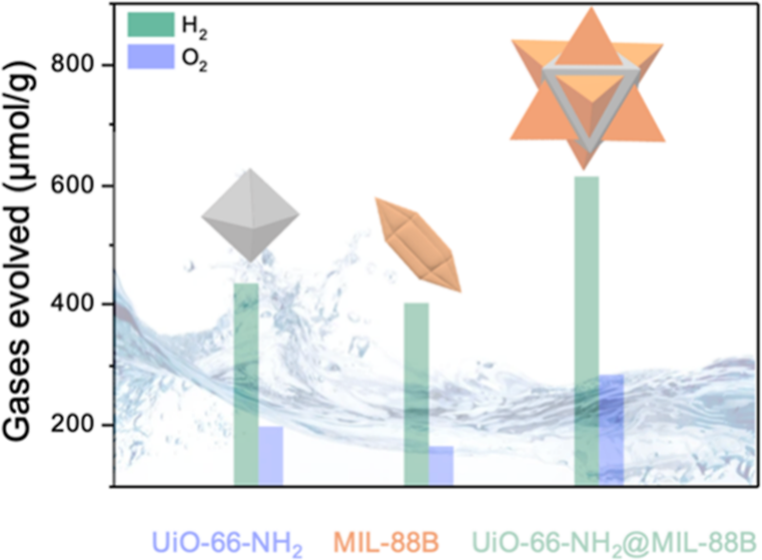

Assembly of different metal–organic frameworks (MOFs) into hybrid MOF-on-MOF heterostructures has been established as a promising approach to develop synergistic performances for a variety of applications. Here, we explore the performance of a MOF-on-MOF heterostructure by epitaxial growth of MIL-88B(Fe) onto UiO-66(Zr)–NH_2_ nanoparticles. The face-selective design and appropriate energy band structure alignment of the selected MOF constituents have permitted its application as an active heterogeneous photocatalyst for solar-driven water splitting. The composite achieves apparent quantum yields for photocatalytic overall water splitting at 400 and 450 nm of about 0.9%, values that compare much favorably with previous analogous reports. Understanding of this high activity has been gained by spectroscopic and electrochemical characterization together with scanning transmission and transmission electron microscopy (STEM, TEM) measurements. This study exemplifies the possibility of developing a MOF-on-MOF heterostructure that operates under a Z-scheme mechanism and exhibits outstanding activity toward photocatalytic water splitting under solar light.

## Introduction

As an ideal energy vector, hydrogen is nowadays considered an alternative to the use of fossil fuels.^1−3^ Accordingly, solar-driven hydrogen production from water is envisioned as one of the cost-efficient and sustainable technologies for this purpose.^3−7^ For instance, some European countries have already started generating green hydrogen by coupling solar photovoltaics and electrolysis technologies. In this context, a more economical while less mature technology to produce hydrogen is solar photocatalysis.^6,7^ Inorganic semiconductors have been studied for about 50 years as photocatalysts for hydrogen evolution reaction (HER) in the presence of sacrificial agents as electron donors and also for the more appealing while more thermodynamically and kinetically demanding overall water splitting (OWS) into H_2_ and O_2_.^6^ Regardless of the excellent achievements made in this area, much effort is under development to find suitable materials for practical applications.^6,8^

This field expanded with the development of metal–organic frameworks (MOFs) as visible-light-responsive materials.^7^ Their unique chemical versatility permits flexibility on metal and ligand constituent selection, thus resulting in a broad range of electronic properties interesting for photocatalytic processes. In 2010, García and co-workers reported a pioneering study on the use of MOFs as photocatalysts for the HER in the presence of sacrificial electron donors under UV–vis irradiation.^9^ Since then, the number of examples showing the potential use of MOFs in this field has significantly increased,^7^ with most of these photocatalysts being constituted by individual MOF structures that can be modified with cocatalysts or other active materials to form composites with enhanced activity.^10−13^ More recently, the possibility of using MOF heterostructures as photocatalysts for HER has attracted interest in the field and is exemplified by the MIL-167@MIL-125(Ti)–NH_2_ system reported by Kampouri et al.^14^ It is postulated that the established heterojunctions between the MOF counterparts enable more efficient visible light absorption and photoinduced charge separation, processes that favor photocatalytic reactions.

A step forward in the field occurred in 2017, when the possibility to use MOFs as photocatalysts for the OWS in the absence of sacrificial agents was demonstrated, using Ni(II) ions coordinated to the amino group of MIL-53(Al)–NH_2_.^15^ Later, other studies focused on the use of Ti-based MOFs such as MIL-125(Ti)–NH_2_^16^ or IEF-11,^17^ mixed-metal MOFs such as UiO-66(Zr/Ce/Ti),^18^ and phosphonate- or porphyrin-based MOFs such as PCN-222^19^ or MIL-173(Zr/Ti).^7,20,21^ Very recently, some of us have reported the development of a core–shell photocatalyst based on UiO-66(Zr)–NH_2_ and UiO-66(Ce) showing an improved activity for OWS under simulated sunlight irradiation compared to the state-of-the-art.^22^ This example reinforces the hypothesis that an epitaxial contact between MOF counterparts in hybrid systems is an adequate strategy for the preparation of efficient photocatalysts for solar-driven water splitting.

Regardless of these precedents, the number of studies showing the potential of hybrid MOF heterostructures for photocatalytic water splitting remains scarce. In the field of photocatalysis using inorganic or carbon-based heterojunctions among others,^23^ it is well-established that improving the interfacial contact between their counterparts increases the efficiency of both interfacial charge transfer and separation and, therefore, boosts the resulting photocatalytic activity. Several synthetic procedures have been reported for the preparation of core–shell structures for this purpose, and the topic has been critically revised.^24^

In this context, engineered hybrid MOF nanostructures with controlled nanocrystal interfaces are also desired to improve their overall electronic and optical properties.^7^ Advantageously, the development of MOF nanohybrids has experienced a recent advance initiated with the pioneering example of core–shell MOFs established by Kitagawa et al. in 2009.^25^ Since then, sophisticated MOF-on-MOF architectures have been developed by epitaxial growth,^26,27^ allowing not only the combination of individual MOF characteristics but also resulting in synergistic performances toward sorption selectivity,^28^ gas storage,^29^ catalysis,^30,31^ drug delivery,^32^ and sensing.^33^ Regardless of few reports showing the potential use of MOF-on-MOF heterostructures such as MIL-167@MIL-125(Ti)–NH_2_^14^ or UiO-66(Zr)–NH_2_@UiO-66(Ce),^22^ the possibility of developing MOF-on-MOF heterostructures by hetero- or epitaxial growth strategies that would favor the interface contact between components to enhance the resulting photocatalytic activity for water splitting has not been yet reported.

Herein, we explore the performance of a MOF-on-MOF heterostructure prepared by epitaxial face-selective growth of MIL-88B(Fe) on UiO-66(Zr)–NH_2_. This hybrid material was evaluated as photocatalyst for water splitting reactions under simulated sunlight irradiation including HER, oxygen evolution reaction (OER), and OWS. Insights on the origin of the improved photocatalytic activity and reaction mechanism were obtained by different spectroscopic techniques such as transient absorption spectroscopy (TAS), time-resolved photoluminescence (TRPL) spectroscopy, electrochemical impedance spectroscopy (EIS), and scanning transmission electron microscopy (STEM) analysis coupled to an energy-dispersive X-ray (EDX) detector.

## Results and Discussion

**Figure 1:**
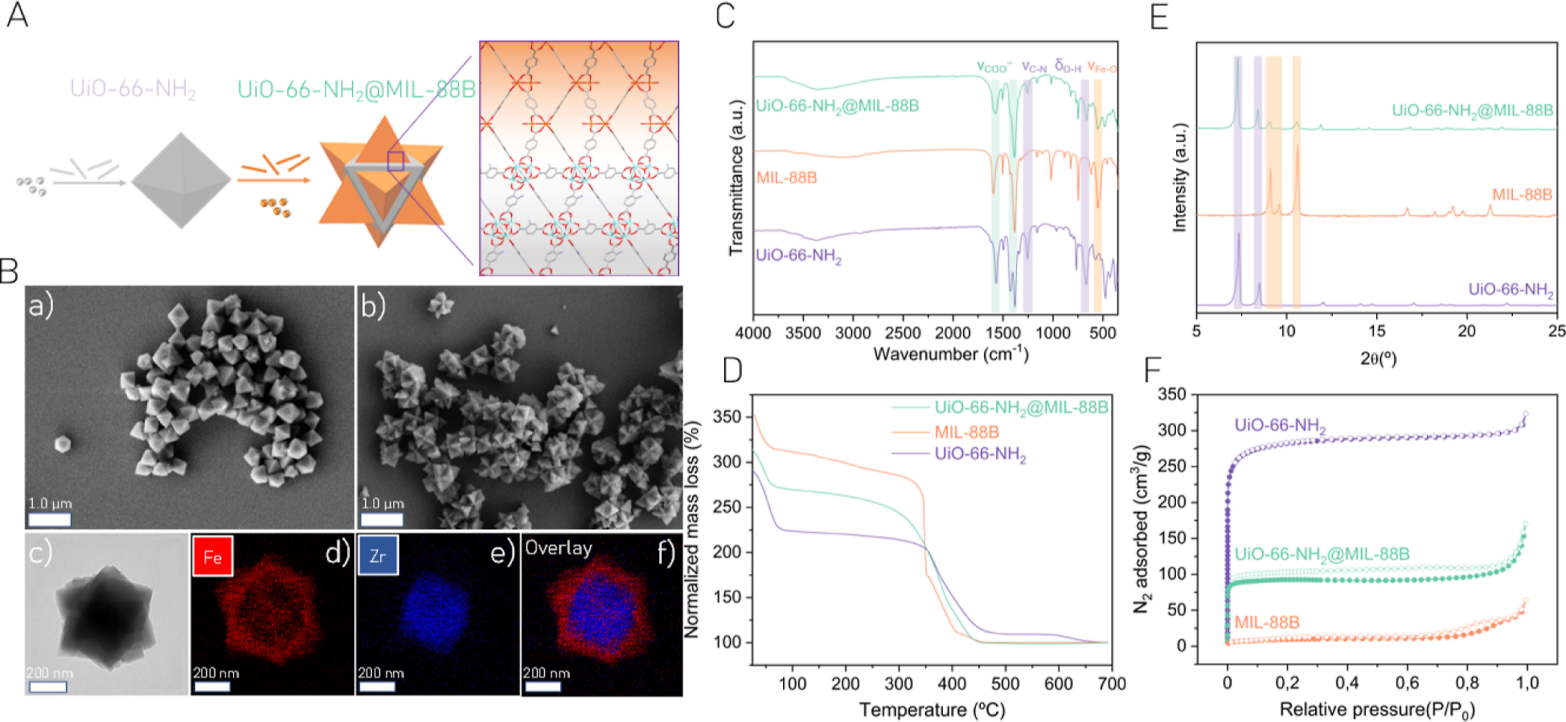
(A) Scheme of the two-step synthesis to obtain hybrid UiO-66(Zr)–NH_2_@MIL-88B(Fe) NPs and crystal matching view at the interface between the (111) plane of UiO-66(Zr)–NH_2_ and the (001) plane of MIL-88B(Fe). (B) SEM images of (a) UiO-66(Zr)–NH_2_ and (b) UiO-66(Zr)–NH_2_@MIL-88B(Fe) NPs, (c) High resolution (HR)-TEM image and (d,e,f) EDX mapping of UiO-66(Zr)–NH_2_@MIL-88B(Fe) NPs. (C) Fourier-transform infrared (FT-IR) spectra. (D) Thermogravimetric analyses (TGA) profiles. (E) XRPD patterns. (F) N_2_ sorption isotherms of UiO-66(Zr)–NH_2_, MIL-88B(Fe), and UiO-66(Zr)–NH_2_@MIL-88B(Fe) materials.

The preparation of the UiO-66(Zr)–NH_2_@MIL-88B(Fe) MOF-on-MOF hybrid structure was developed by direct epitaxial growth of carboxylate-based MIL-88B(Fe) selectively grown onto preformed UiO-66(Zr)–NH_2_ nanoparticles (NPs) (Figure 1A). First, uniform size NPs of UiO-66(Zr)–NH_2_ (473 ± 52 nm, Figure S1) were obtained by reacting 2-aminoterephthalic acid with zirconyl chloride octahydrate via a solvothermal procedure in dimethylformamide (DMF) and comodulated by acetic acid and triethylamine, adapting a procedure from the literature.^34^ Morphology and phase purity of the octahedral NPs were confirmed, respectively, by field emission scanning electron microscopy (FESEM) and X-ray powder diffraction (XRPD) [Figures 1B(a) and S2]. Then, the UiO-66(Zr)–NH_2_@MIL-88B(Fe) heterostructure was obtained by the solvothermal reaction of UiO-66(Zr)–NH_2_ NPs with terephthalic acid and iron(III) chloride in DMF via a solvothermal process modified from the literature.^35^ Triethylamine was used to promote the epitaxial growth, avoiding slow nucleation of MIL-88B(Fe). As control, individual MIL-88B(Fe) material was synthesized under similar conditions (see Supporting Information for details).

After epitaxial growth, hybridized star-shaped UiO-66(Zr)–NH_2_@MIL-88B(Fe) NPs were obtained as determined from FESEM and TEM [Figure 1B(b,c)]. It is observed that hexagonal MIL-88B(Fe) MOF crystals are specifically grown on the facets of octahedral UiO-66(Zr)–NH_2_ NPs, as expected for the chemical connection and crystallographic matching between the MIL-88B(Fe) (001) plane with the cubic UiO-66(Zr)–NH_2_ (111) plane [reported 2D lattice parameters of 14.416 and 14.637 Å, for MIL-88B(Fe) and UiO-66(Zr)–NH_2_, respectively].^32,35^ The structural organization in the hybrid was confirmed by elemental analysis using HRTEM [Figure 1B(d–f)] to localize the metals, zirconium being positioned at the core and iron on the tips of the star-shaped particles.

Composition in the hybrid UiO-66(Zr)–NH_2_@MIL-88B(Fe) heterostructure was assessed by different techniques, including inductively coupled plasma mass spectroscopy (ICP–MS) and proton nuclear magnetic resonance spectroscopy (^1^H NMR) to, respectively, determine the Fe/Zr molar content and the ligand molar ratio between terephthalic acid and 2-aminoterephthalic acid from digested samples. At this point, the possible presence of missing clusters and/or linkers defects in the UiO-66(Zr)–NH_2_ MOF was considered,^36,37^ revealing no evidence of metal cluster defects as deduced by XRPD and pore size distribution analysis (Figures S2 and S3). Missing linkers were initially identified by TGA of UiO-66(Zr)–NH_2_ (Figure S4 and Table S1), which reveals a lower organic content in the experimental profile compared to that in the theoretical material. The percentage of missing linker defects was estimated at 24% by TGA.^38^ The ICP–MS and data (Figure S5 and Table S2) were then in good agreement after taking into account the missing linker defects, giving an average mass ratio of 57/43 for MIL-88B(Fe) and UiO-66(Zr)–NH_2_, respectively.

The hybrid UiO-66(Zr)–NH_2_@MIL-88B(Fe) NPs display characteristic IR bands as well as thermal degradation profiles of individual components (Figures 1C,D and S6), and phase purity was confirmed by XRPD (Figure 1E). In addition, the distinctive solvent-responsive dynamic character of the MIL-88B(Fe) structure was retained in the hybrid material (Figure S7).^39^ The sorption capacity of the MOF heterostructure was investigated by N_2_ (Figures 1F and S8) and CO_2_ (Figure S9) sorption isotherms at 77 and 273 K, respectively, resulting in the combination of single MOF performances in both cases.

**Figure 2:**
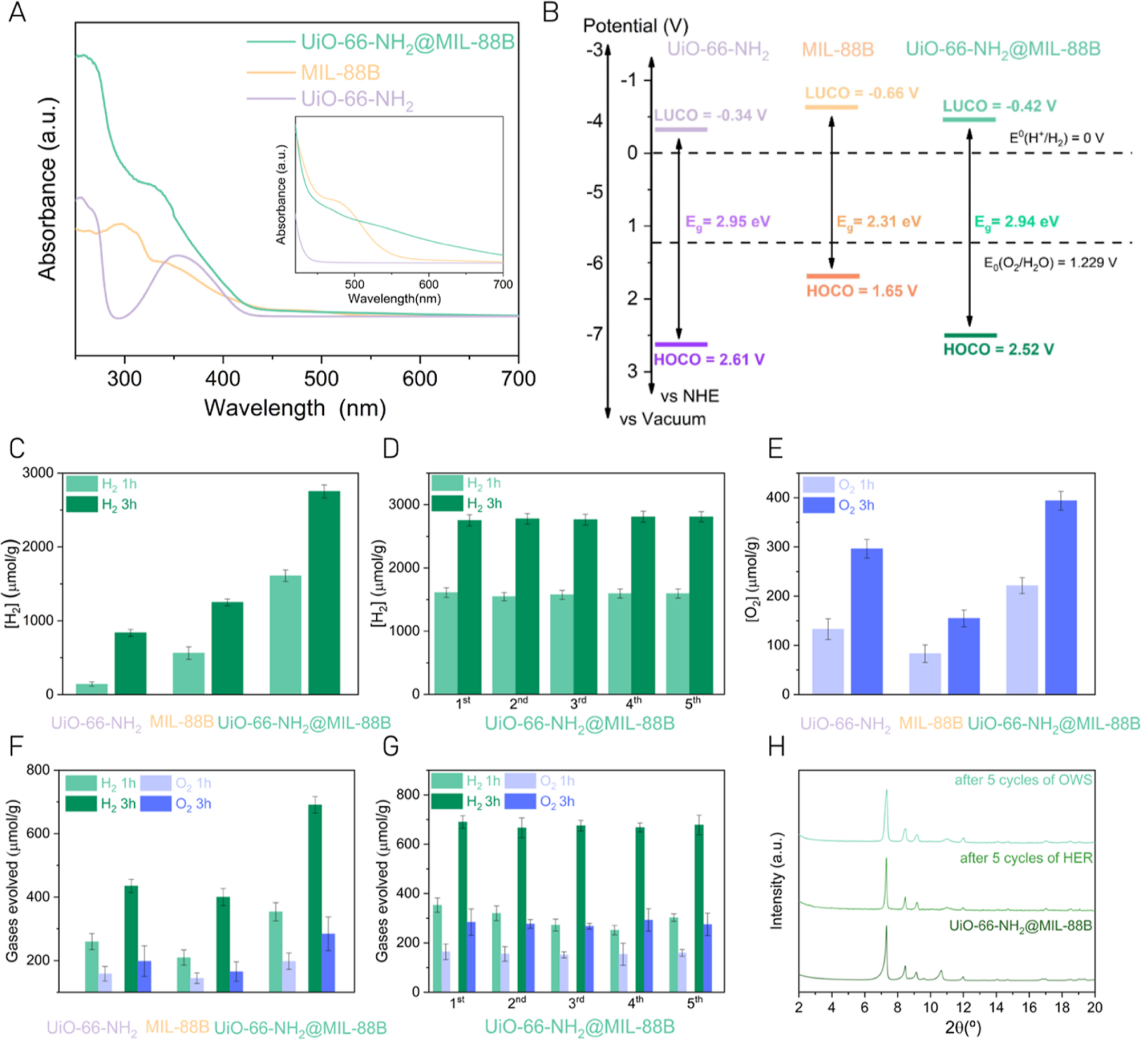
(A) UV–vis DRS diagram of UiO-66(Zr)–NH_2_, MIL-88B(Fe), and UiO-66(Zr)–NH_2_@MIL-88B(Fe), with the inset displaying the region between 425 and 700 nm. (B) Diagram of energy band levels. (C) Photocatalytic HER at 1 and 3 h of reaction time and (D) UiO-66(Zr)–NH_2_@MIL-88B(Fe) HER performance for five consecutive cycles. Reaction conditions: photocatalyst (5 mg), H_2_O (8 mL), MeOH (2 mL), and 35 °C. (E) Photocatalytic OER at 1 and 3 h of reaction time. Reaction conditions: photocatalyst (10 mg), H_2_O (20 mL), Na_2_S_2_O_8_ (700 mg), and 35 °C. (F) Photocatalytic OWS at 1 and 3 h of reaction time and (G) UiO-66(Zr)–NH_2_@MIL-88B(Fe) OWS performance for five consecutive cycles. Reaction conditions: photocatalyst (10 mg), H_2_O (20 mL), and 35 °C. (H) XRPD patterns of UiO-66(Zr)–NH_2_@MIL-88B(Fe) before and after five consecutive cycles of photocatalytic HER and OWS. Note: Photocatalytic reactions under simulated sunlight irradiation use a Hg–Xe lamp 150 W through an AM 1.5G filter (320 mW/cm^2^).

X-ray photoelectron spectroscopy (XPS) and UV–Vis spectroscopic analyses were used to characterize the elements and their oxidation state within the hybrid heterostructure as compared to the individual UiO-66(Zr)–NH_2_ and MIL-88B(Fe) solids (Figures S10–14). XPS revealed the presence of Zr(IV) and Fe(III) in the MOF-on-MOF structure (Figure S13), whereas UV–Vis diffuse reflectance spectroscopy (DRS) showed the characteristic bands of both MOF counterparts, together with the appearance of an absorption feature in the visible region from *ca*. 550 to 650 nm, which is associated with the electronic contact created between the two MOF counterparts (Figures 2A and S14).^22^ UV–Vis DRS data were used to estimate the optical band gaps of the materials by representing the Tauc plot graphs (Figure S15), resulting in calculated band gaps of 2.95, 2.31, and 2.94 eV, respectively, for UiO-66(Zr)–NH_2_, MIL-88B(Fe), and UiO-66(Zr)–NH_2_@MIL-88B(Fe) materials.

To evaluate the capabilities of the hybrid heterostructure as a photocatalyst for water splitting reactions as compared to the individual MOFs, the energy band level diagrams of the solids were analyzed. The energy values of the highest occupied crystal orbital (HOCO) of the solids were estimated by means of HOCO-XPS,^40^ whereas the lowest unoccupied crystal orbital (LUCO) energy data were determined from the optical band gap and the HOCO values (Figure S15). As depicted in Figure 2B, the individual and the hybrid MOF-based photocatalysts exhibit adequate thermodynamic energy levels for HER, OER, and OWS processes under solar light radiation.

### Photocatalytic Activity

Initially, the UiO-66(Zr)–NH_2_@MIL-88B(Fe) heterostructure and the corresponding single components were evaluated for the individual photocatalytic HER or OER reactions (Figure 2C–E). Although with different efficiencies, the three materials were active photocatalysts for these processes, in agreement with the estimated band energy levels of the MOFs that meet the thermodynamic requirements for water splitting into H_2_ and/or O_2_. The higher activity of MIL-88B(Fe) vs. UiO-66(Zr)–NH_2_ for the HER (1250 *vs*. 836 μmol g^–1^ at 3 h, respectively) is attributed to both its lowest optical band gap and a more negative LUCO value that supports the proton reduction reaction under simulated sunlight irradiation. In contrast, UiO-66(Zr)–NH_2_ shows higher activity for the OER, a fact that is associated with the more positive HOCO value that would favor water oxidation. Importantly, the UiO-66(Zr)–NH_2_@MIL-88B(Fe) heterostructure exhibits the highest activity for both HER and OER compared to the individual MOFs (Figure 2C,E). A control experiment using a physical mixture of UiO-66(Zr)–NH_2_ and MIL-88B(Fe) with similar proportions to those present in the heterostructure [57/43 mass ratio of MIL-88B(Fe) and UiO-66(Zr)–NH_2_ respectively] resulted in an activity similar for the photocatalytic HER to the one achieved using the individual MOFs (1310 ± 230 μmol g^–1^ after 3 h). It is found that the heterostructure maintains its photocatalytic activity for the HER during five consecutive cycles (Figure 2D) while retaining crystal integrity, as deduced by XRPD (Figure 2H). In addition, inductively coupled plasma optical emission spectroscopy (ICP-OES) analyses revealed only a low iron leaching (0.3 wt % of the total Fe present on the MOF-on-MOF photocatalyst) and no detectable zirconium leaching after catalyst reuse up to five cycles. The use of the heterostructure as heterogeneous photocatalyst during HER resulted in apparent quantum yields (AQYs) of 6.5, 6.5, 1.7, and 1.5%, respectively, at 400, 450, 500, and 550 nm.

The photocatalysts were further tested for the challenge of the OWS reaction under simulated sunlight irradiation, with the heterostructure resulting in higher values as compared to the individual MOFs (Figure 2F) or a physical mixture (463 and 201 μmol g^–1^ of H_2_ and O_2_, respectively, after 3 h), achieving quasi-stoichiometric amounts of evolved gases. The influence of the irradiation conditions on the photocatalytic activity of the heterostructure was investigated (Figure S16), determining that the highest activity achieved in terms of the amount of evolved gases with respect to the irradiance corresponds to the use of UV light. This observation agrees with the highest energy of UV photons compared to that of visible ones. Interestingly, similar activity was achieved using UV–vis or simulated sunlight irradiations, while the achieved photocatalytic activity under visible light irradiation (>400 nm) corresponds to more than about 25% that of simulated sunlight irradiation. A photocatalytic control experiment under simulated sunlight irradiation using labeled H_2_^18^O and the heterostructure revealed the formation of ^18^O_2_ evidencing the photocatalytic oxidation of water to O_2_ (Figure S17). Furthermore, the heterostructure was reused five consecutive times without significant loss of activity (Figure 2G). This optimal recycling was achieved with retention of crystal integrity and morphology, based, respectively, on XRPD (Figure 2H) and SEM (Figure S18) analyses, whereas low (0.52 wt %) iron and negligible zirconium leaching was determined by ICP-OES analysis of the reaction medium. The observed higher iron leaching during photocatalytic OWS compared to that during the HER is attributable to some extent to the lower chemical stability of UiO-66(Zr)–NH_2_@MIL-88B in water compared to the use of a MeOH and water mixture (see Table S3 for details). Moreover, this higher iron leaching may be a consequence of the more thermodynamically demanding H_2_O-oxidation reaction to O_2_ during photocatalytic OWS compared to photocatalytic proton reduction to H_2_ during HER, as previously proposed in related studies.^41^ The estimated AQYs achieved using UiO-66(Zr)–NH_2_@MIL-88B(Fe) as photocatalyst during the OWS at 400, 450, 500, and 550 nm were 0.91, 0.86, 0.19, and 0.14%, respectively.

We hypothesize that the enhanced photocatalytic activity of the heterostructure is associated with the epitaxial interface contact between UiO-66(Zr)–NH_2_ and MIL-88B(Fe), which is expected to increase the efficiency of photoinduced charge separation, thus resulting in the observed photocatalytic activity. This hypothesis will be discussed in detail. One may consider that the presence of missing-linker defects in the heterostructure located at the UiO-66(Zr)–NH_2_ counterpart may influence its photocatalytic activity.^42^ However, we have experimentally observed that the defectivity of the former UiO-66(Zr)–NH_2_ NPs is retained in the hybrid system after the epitaxial reaction (Figure S19). In this scenario, the different photocatalytic performance observed is related to the intrinsic benefits of the UiO-66(Zr)–NH_2_@MIL-88B heterojunction improving the photoinduced charge separation efficiency, as evidenced by (photo)electrochemical and spectroscopic analyses such as TAS or photoluminescence (PL) spectroscopy.

Table 1 summarizes the achieved photocatalytic activity of UiO-66(Zr)–NH_2_@MIL-88B(Fe) during the OWS with related precedents using MOF-based materials.^16,17,19,21,22,41−44^ As it can be seen, the photocatalytic activity of UiO-66(Zr)–NH_2_@MIL-88B(Fe) compares favorably (H_2_ and O_2_ production of 690 and 279 μmol g^–1^ in 3 h, respectively; AQY at 400 and 450 nm of *ca*. 0.9%) with previous reports under similar reaction conditions.

**Table 1:** Summary of Photocatalytic Activities Reported Using MOFs for Solar-Driven OWS

photocatalyst	reaction conditions	H_2_ and O_2_ production (μmol g^–1^)	AQY	ref
UiO-66(Zr)–NH_2_@MIL-88B(Fe)	photocatalyst (10 mg), H_2_O (20 mL), UV–Vis (150 W Hg–Xe lamp through an AM 1.5G filter (320 mW/cm^2^), and 35 °C	690 and 279 in 3 h	0.91 and 0.86% at 400 and 450 nm	this work
		1108 and 482 in 5 h		
UiO-66(Zr)–NH_2_	photocatalyst (10 mg), H_2_O (20 mL), UV–Vis (150 W Hg–Xe lamp through an AM 1.5G filter (320 mW/cm^2^), and 35 °C	682 and 274 in 5 h		this work
UiO-66(Zr)–NH_2_	photocatalyst (10 mg), H_2_O (20 mL), simulated sunlight irradiation (150 W Hg–Xe lamp through an AM 1.5G filter, 220 mW/cm^2^), and 35 °C	450 and 160 in 5 h	0.06 and 0.04% at 400 and 450 nm	(42)
UiO-66(Zr)–NH_2_@UiO-66(Ce)	photocatalyst (10 mg), H_2_O (20 mL), simulated sunlight irradiation (150 W Hg–Xe lamp through an AM 1.5G filter), and 35 °C	375 and 170 in 22 h	0.034% at 400 nm	(22)
UiO-66(Ce)–NH_2_	photocatalyst (20 mg), H_2_O (20 mL), simulated sunlight irradiation (150 W Hg–Xe lamp equipped with an AM 1.5G filter), and 35 °C	208 and 80 in 22 h		(41)
UiO-66(Zr/Ce/Ti)	photocatalyst (20 mg), H_2_O (20 mL), and visible light irradiation (150 W Hg–Xe lamp with a λ > 450 nm cutoff filter)	210 and 70 in 22 h		(43)
MIL-125(Ti)–NH_2_	photocatalyst (20 mg), H_2_O (20 mL), 35 °C, and solar simulator (1 sun)	83 and 29 in 22 h		(16)
Ti-MOF: IEF-11	photocatalyst (10 mg), H_2_O (20 mL), simulated sunlight irradiation (150 W Xe–Hg lamp, 1.5 AM filter), and 35 °C	260 and 107 in 22 h		(17)
liposome-MOF hybrid	photocatalyst solution (10 mL) and redox relays,^a^ H_2_O (20 mL), and LED light	836 and 418 in 72 h	1.5% at 436 nm	(44)
MIL-173(Zr/Ti)-40	photocatalyst (10 mg), H_2_O (20 mL), simulated sunlight irradiation (Xe–Hg lamp 150 W, 1.5 AM filter), and 35 °C	381 and 145 in 22 h	0.11% at 450 nm	(21)
PCN-222(Zn)	photocatalyst (20 mg), H_2_O (20 mL), and simulated sunlight irradiation (1 sun)	340 and 30 in 22 h		(19)

a Fe^3+/^Fe^2+^, tetrachlorobenzoquinone/tetrachlorobenzohydrosemiquinone.

Caution should be taken when comparing the photocatalytic results carried out using different reactor setups (light intensity, spectral range, reactor design, photocatalyst amount, stirring speed, etc.) in terms of μmol g^–1^ of photocatalyst. Another common parameter employed in photocatalysis to compare the efficiency of these solids during the OWS is the AQY, which considers the μmol production of H_2_ or O_2_ as a function of the light intensity for monochromatic light. In this context, UiO-66(Zr)–NH_2_@MIL-88B(Fe) exhibits much higher AQYs under visible light irradiation than previous related studies using UiO-66(Zr)–NH_2_ from modulated synthesis with acetic acid (0.06% at 400 nm),^42^ UiO-66(Zr)–NH_2_@UiO-66(Ce) (0.034% at 400 nm),^22^ and even a mixed-metal porphyrin-based MOF termed MIL-173(Zr/Ti)-40 (0.11% at 450 nm).^21^ Notably, considering that UiO-66(Zr)–NH_2_@MIL-88B(Fe) is a noble metal-free hybrid structure, the achieved AQYs compares favorably with a precedent using a liposome-based MOF containing Pt-porphyrin, [Ru(2,2-bipyridine)_3_]^2+^ and Ir-bipyridine catalytic centers combined with homogeneous redox relays species, with the highest AQY of 1.5 ± 1% at 436 nm.^44^

### Photoinduced Charge Carrier Generation and Redox Sites^31^

**Figure 3:**
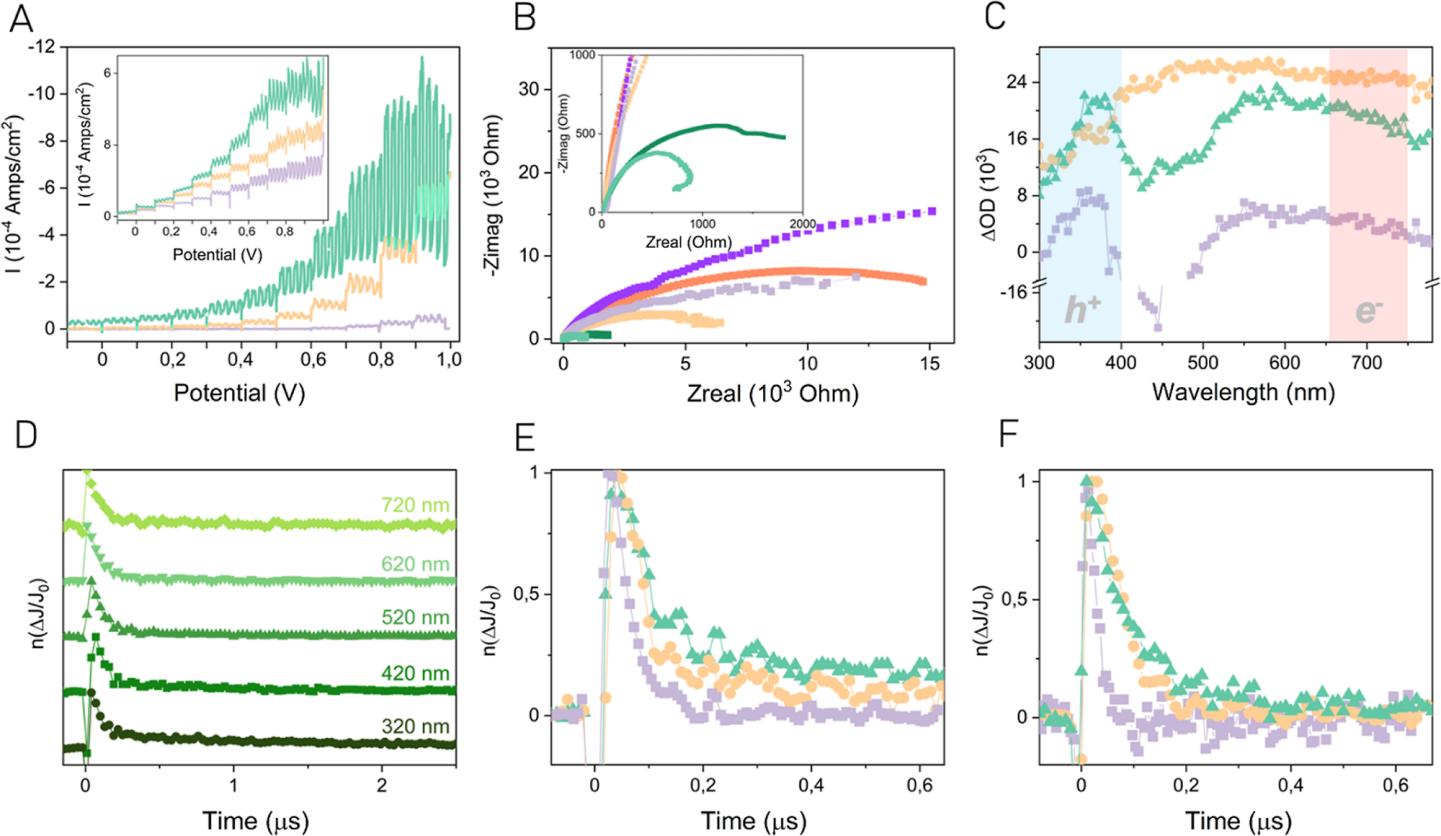
(A) Photocurrent response of UiO-66(Zr)–NH_2_ (purple), MIL-88B(Fe) (orange), and UiO-66(Zr)–NH_2_@MIL-88B(Fe) (green) materials supported on a carbon glass electrode in a deoxygenated TBAPF_6_ (0.1 M) acetonitrile (ACN) solution under simulated sunlight irradiation (Hg–Xe lamp 150 W through an AM 1.5G filter, 320 mW/cm^2^). Inset: results in the presence of MeOH. (B) EIS of the samples at −0.7 V for UiO-66(Zr)–NH_2_, MIL-88B(Fe), and UiO-66(Zr)–NH_2_@MIL-88B(Fe) without irradiation conditions (dark purple, orange, and green profiles, respectively) or under simulated sunlight irradiation (light purple, orange, and green profiles, respectively). Inset: zoom-in of the region between 0 and 2000 Ohm. (C) TAS of UiO-66(Zr)–NH_2_ (purple squares), MIL-88B(Fe) (orange spheres), and UiO-66(Zr)–NH_2_@MIL-88B(Fe) (green triangles) under argon and in ACN, recorded 30 ns after 266 nm laser excitation. Light blue and red regions in the light spectrum of TAS denote the expected position of the photogenerated holes and electrons, respectively. (D) Normalized temporal profile of the transient signals of UiO-66(Zr)–NH_2_@MIL-88B(Fe) at several wavelengths. (E) Normalized temporal profile of the transient signals recorded at 320 nm and (F) 720 nm after excitation at 266 nm for UiO-66(Zr)–NH_2_ (purple squares), MIL-88B(Fe) (orange spheres), and UiO-66-NH_2_@MIL-88B(Fe) (green triangles).

To gain some understanding about the higher photocatalytic activity observed in the hybrid UiO-66(Zr)–NH_2_@MIL-88B(Fe) material compared to that of the individual MOFs, the photoinduced charge carrier generation was investigated. For this purpose, photoelectrochemical measurements, TAS, TRPL, and STEM–EDX analyses were carried out. Initially, the transient photocurrent response of the MOFs under simulated sunlight irradiation was measured. Figure 3A shows that the highest current intensity of the materials is achieved for the hybrid UiO-66(Zr)–NH_2_@MIL-88B(Fe) solid supported on fluorine-doped tin oxide (FTO) as the working electrode polarized at +0.9 V. Similar measurements performed in the presence of MeOH result in a great increase of the recorded current intensity. This observation agrees with the role of the sacrificial electron donor of MeOH that becomes oxidized by the photogenerated holes of the MOF, thus increasing the population of electrons, which results in an increase of the photocurrent intensity. Interestingly, the order of photocurrent intensity in the presence of MeOH follows the trend UiO-66(Zr)–NH_2_@MIL-88B(Fe) > MIL-88B(Fe) > UiO-66(Zr)–NH_2_, which is the same as the photocatalytic HER activity.

The three materials were further characterized by EIS analyses (Figure 3B). The lower Nyquist radii observed when using the heterostructure with respect to the individual MOFs indicate its lower charge transfer resistance. Analogous EIS measurements upon simulated sunlight irradiation of the three photocatalysts revealed that the UiO-66(Zr)–NH_2_@MIL-88B(Fe) hybrid exhibits the highest reduction of Nyquist radii, which are compared to measurements under dark conditions, thus indicating a higher decrease in charge transfer resistance during the photocatalytic reaction. EIS results are in good agreement with the observed order during the photocurrent measurements and photocatalytic activity. Overall, transient photocurrent measurements and EIS analyses indicate that the hybrid MOF-on-MOF exhibits the highest photoinduced charge separation efficiency of the series, a situation that agrees with its highest photocatalytic activity compared to individual MOFs.

TAS on the microsecond timescale was used to monitor the photoexcited states of the materials. For this purpose, a series of Ar-purged acetonitrile (ACN) suspensions of the MOFs with the same absorbance (*ca*. 0.35 au) were prepared. TAS studies were carried out at 266 and 355 nm pulsed laser excitation, and similar results were obtained (Figures S20–S22). Essentially, upon excitation at 266 nm, the transient spectra in the microsecond timescale of UiO-66(Zr)–NH_2_, MIL-88B(Fe), and UiO-66(Zr)–NH_2_@MIL-88B(Fe) are characterized by continuous absorption spanning the whole UV–vis wavelength range (Figure 3C). The difference in the case of UiO-66(Zr)–NH_2_ was the spectrum at the shortest time available to our nanosecond laser flash system (15 ns) that exhibited a negative signal at λ_max_ 450 nm (Figure 3C, purple squares) corresponding to the residual PL from this MOF due to the presence of 2-aminoterephthalic acid. This emission is also observed as a less intense absorption at 450 nm in the UiO-66(Zr)–NH_2_@MIL-88B(Fe) spectra recorded at short delay times (<50 ns), disappearing at longer times, thereby resulting in a flat transient absorption (Figure S20). For the three samples, the signal temporal profiles at different wavelengths were coincident (Figure 3C), meaning that the kinetics are constant throughout the wavelength range and the transient species decay by recombination. In the case of UiO-66(Zr)–NH_2_@MIL-88B(Fe), the temporal signal profiles exhibit two regimes (Figure 3D), one very fast, decaying in a few tens of nanoseconds corresponding to 85% of the total initial intensity, and the other a relatively longer residual signal, extending a few microseconds.

These two regimes could be rationalized considering geminal transient annihilation for fast decay and annihilation after migration for longer decay kinetics. Information about the nature of the transient spectra was gained by monitoring the influence of MeOH and O_2_ as hole and electron quenchers, respectively. The influence of these two quenchers was mostly reflected in changes in the initial signal intensity, meaning that in both cases, the quenching occurs faster than the temporal response of our laser flash photolysis setup, affecting most probably the fast kinetics. The three MOFs under study behave identically with the quenchers, except for UiO-66(Zr)–NH_2_@MIL-88B(Fe) in the case of MeOH quenching in the red spectral region (650–750 nm), which will be commented on separately. For each of the three MOFs, the influence of the quenchers was opposite in the UV-blue region and the red spectral zone. It was observed that MeOH decreased the intensity of the initial signal in the UV-blue wavelength range and increased the intensity of the initial signal in the red spectral zone. An opposite behavior was observed in the three MOFs for O_2_ quenching, *i.e.*, an increase of the signal intensity in the blue part, which led to a decrease in the initial signal intensity in the red part of the spectra (Figures S21 and S22). These quenching patterns are compatible with the photogeneration of electrons and holes, with holes contributing to a larger extent to the transient absorption in the UV-blue spectral zone and electrons being responsible for a larger absorption in the red part of the transient spectrum (Figure 3C). The only quenching behavior that does not follow this pattern of electron/hole separation is the case of UiO-66(Zr)–NH_2_@MIL-88B(Fe) in MeOH in the red spectral zone, for which also a decrease of the initial signal intensity was observed, meaning that for the heterojunction, holes also contribute to the red side of the spectrum. TAS data agree with the photoinduced generation of electrons and holes, where holes would contribute more to the transient signal at shorter wavelengths (300–400 nm) and electrons exhibit larger contribution in the 600–800 nm range. Besides supporting the photoinduced ligand-to-metal electron transfer, the most relevant information provided by TAS regarding the operation of the MOF-on-MOF heterojunction was the signal lifetime that becomes notably increased as compared to the individual components, particularly UiO-66(Zr)–NH_2_, which has the shortest transient lifetimes. This situation observed for the heterostructure agrees with partial suppression of charge carrier recombination and promotion of their spatial separation, which, thus, improve its photocatalytic activity with respect to that of the individual MOFs. Figure 3E,F display a comparison of the temporal profile of the transient signals recorded at 320 and 720 nm for the three catalysts upon 266 nm excitation. These transient signals at 320 and 720 nm are selected provided their correspondence with the domains associated with the presence of holes and electrons, respectively. These regions were evidenced using MeOH and O_2_ as hole and electron trapping agents, respectively (Figure S21c). This extended transient lifetime in the microsecond timescale would be a consequence of the effective charge migration through the heterojunction, beneficial for the OWS as experimentally observed. These observations were further supported by means of TRPL decay kinetic studies upon photoexcitation at 340 or 266 nm using UiO-66(Zr)–NH_2_, MIL-88B(Fe), and UiO-66(Zr)–NH_2_@MIL-88B(Fe) materials suspended in ACN (Figure S23). An important increase of PL lifetime is observed in the case of UiO-66(Zr)–NH_2_@MIL-88B(Fe) with respect to that of the individual MOFs, which is associated with the spatial separation of photogenerated carriers at the interface of UiO-66(Zr)–NH_2_ and MIL-88B(Fe),^45^ in good agreement with TAS conclusions. Note that PL lifetime values below 2 ns are within the limit of detection of our instrument.

**Figure 4:**
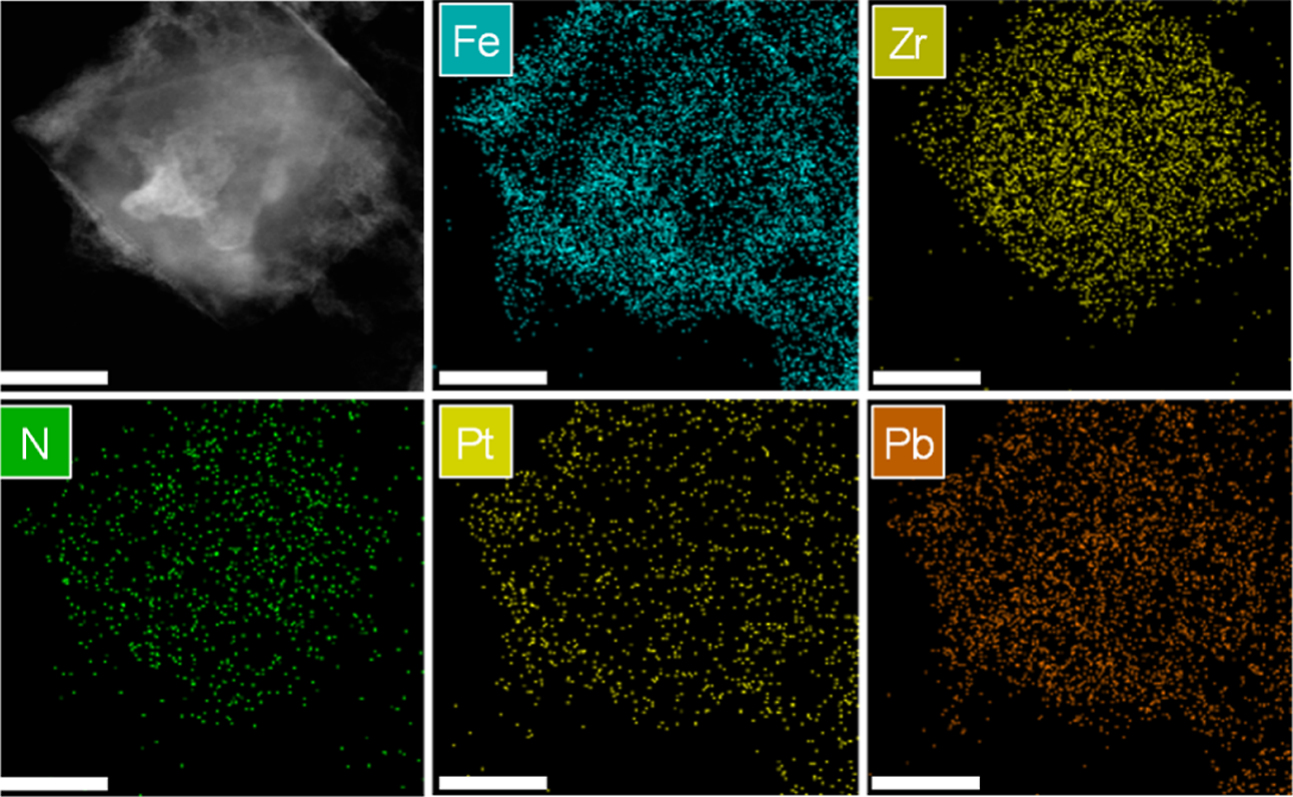
STEM image and elemental EDX mapping for UiO-66(Zr)–NH_2_@MIL-88B(Fe) supported Pt and PbOx. Scale bar: 100 nm. After the photodeposition process, Pt and PbOx NPs were, respectively, deposited on the different MIL-88B(Fe) (reduction sites) and UiO-66-(Zr)–NH_2_ (oxidation sites) parts of the heterojunction.

At this point, additional experiments were carried out to determine the different spatial locations of photogenerated electrons and holes within the hybrid UiO-66(Zr)–NH_2_@MIL-88B(Fe) solid. A general methodology to gain information on the operation of heterojunctions is the so-called selective photodeposition. Charge carrier migration across interfaces leads to the preferential distinctive spatial location of electron and holes in a heterojunction^46^ including MOF heterostructures.^22^ Visualization of the selective location of metal and metal oxide NPs by electron microscopy is firm evidence of the specific role of the components as reducing and oxidizing photocatalysts in a heterojunction. Thus, photodeposition experiments were carried out suspending UiO-66(Zr)–NH_2_@MIL-88B(Fe) in water containing an appropriate soluble transition metal salt precursor for selective deposition in different regions of the heterostructure depending on the nature of the sacrificial agent. In particular, H_2_PtCl_6_ and Pb(NO_3_)_2_ or Co(NO_3_)_2_ were employed for the selective deposition of Pt and PbO_2_ or CoO_*x*_ NPs at the reduction and oxidation sites of UiO-66(Zr)–NH_2_@MIL-88B(Fe), respectively. After photodeposition, the resulting material was analyzed by STEM–EDX, which provides an elemental mapping of the material at a submicrometric resolution. Figure 4 exhibits the preferential location of Pt NPs at the MIL-88B(Fe) component, while PbO_2_ is mainly deposited on the UiO-66(Zr)–NH_2_ parts of the heterojunction. Elemental mapping presented as a STEM–EDX line corroborates the selective location of photodeposited Pt on MIL-88B(Fe) and PbO_2_ or CoO_*x*_ at UiO-66(Zr)–NH_2_ (see Figures S24–S29).

**Figure 5:**
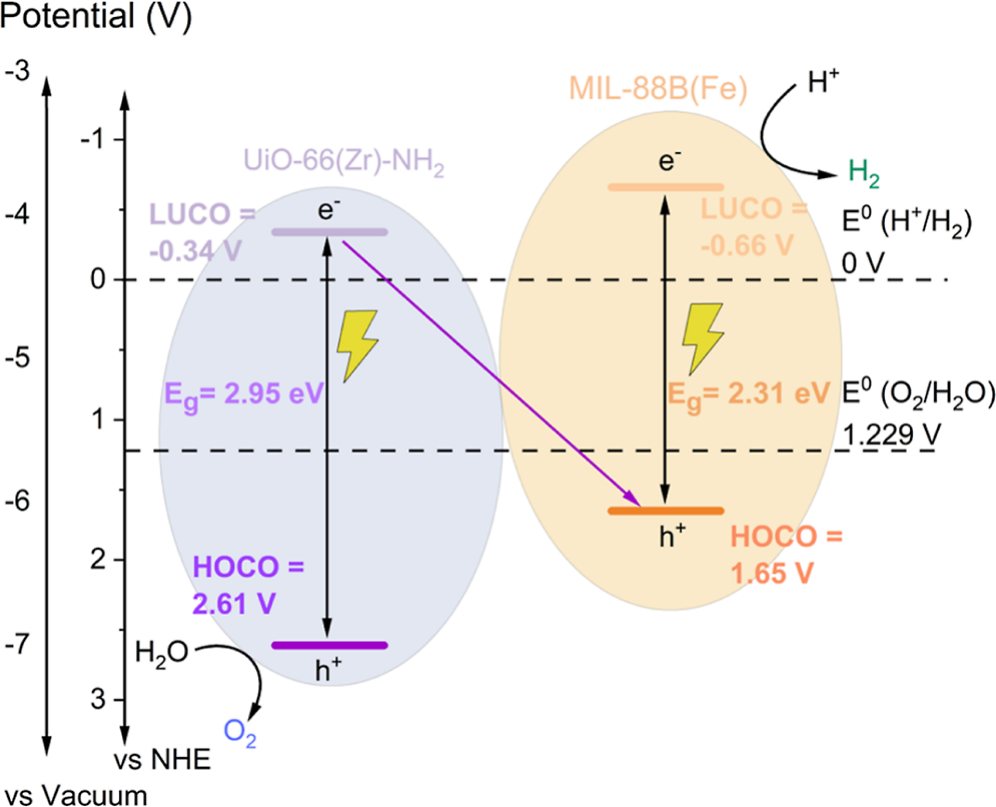
Proposed photocatalytic mechanism of the Z-scheme using UiO-66(Zr)–NH_2_@MIL-88B(Fe).

These observed preferential depositions agree with the performance of the UiO-66(Zr)–NH_2_@MIL-88B(Fe) heterostructure upon irradiation via a Z-scheme mechanism (Figure 5). Specifically, upon irradiation of the UiO-66(Zr)–NH_2_@MIL-88B(Fe) heterostructure, electrons from the HOCO are excited to the LUCO level of each counterpart. Then, photogenerated electrons present in the LUCO of UiO-66(Zr)–NH_2_ recombine with the holes present in the HOCO of MIL-88B(Fe). Therefore, there is a preferential deposition of Pt and PbOx or CoOx NPs within the reductive and oxidative sites of the heterostructure, respectively, namely, MIL-88B(Fe) and UiO-66(Zr)–NH_2._ Therefore, the reductive and oxidative active sites of the heterostructure are located into MIL-88B(Fe) and UiO-66(Zr)–NH_2_, respectively.

In connection with the major active species during photocatalytic hydrogen generation, previous studies using MIL-88B(Fe)^45^ and other related Fe(III)-MOFs solids as photocatalysts have proposed that the major active reducing species are photogenerated reductive Fe(II) sites.^47,48^ In addition, previous transient photocurrent measurements have shown that the heterostructure exhibits the highest current intensity in the series. These current intensities are due to the number of electrons extracted from the solids, and therefore, these values represent in some extent the number of active sites in the photocatalysts.

To gain more insights about the active species and charge carrier migration pathways during photocatalytic experiments, additional *in situ* XPS measurements and photocatalytic trapping experiments were carried out (Figure S30). *In situ* XPS has been previously used to detect changes in the electron density within a heterostructure before and after irradiation with reference C 1s as the calibration peak.^49^ The observation of positive and negative shifts in binding energies is generally ascribed to a decrease or increase of electron density in these elements, respectively, thus resulting in an appropriate tool to gain information about the migration pathway of photogenerated charge carriers. Specifically, the observed binding energies of XPS Fe 2p are shifted toward lower binding energies (*ca*. 0.1 eV) upon irradiation, whereas the binding energies of XPS N 1s and XPS Zr 3d are shifted to more positive values (ca. 0.1 eV). These results agree with the preferential accumulation of photogenerated electrons in Fe(III) elements of MIL-88B(Fe), while photogenerated holes accumulated in Zr(IV) and N elements of the UiO-66(Zr)–NH_2_ counterpart. These observations are in accordance with the operation of the heterojunction under a Z-scheme mechanism.

The role of the active species during photocatalytic water splitting processes was explored by using different concentrations of MeOH or sodium persulfate as trapping agents of photogenerated electrons and holes, respectively. The obtained results indicate that the use of increasing amounts of sodium persulfate in the reaction results in a higher production of evolved molecular O_2_ (Figure S31a), in good agreement with the role of persulfate as the electron acceptor and, thus, lead to quenching of the photogenerated electrons. Similarly, the use of larger amounts of MeOH during the reaction results in a gradual increase of hydrogen production (Figure S31b), evidencing the role of MeOH as a hole trapping agent.

Overall, the higher photocatalytic activity of the UiO-66(Zr)–NH_2_@MIL-88B(Fe) solid is associated with the more efficient photoinduced charge separation that occurs on the hybrid heterostructure and it is related to the selective separation of electrons and holes, respectively, on MIL-88B(Fe) and UiO-66(Zr)–NH_2_ counterparts. These results confirm that the Z-scheme heterojunction is also valid in MOF heterojunctions (Figure 5).

## Conclusions

Herein, we have shown the preparation of a MOF-on-MOF heterostructure via heteroepitaxial growth of selected hexagonal MIL-88B(Fe) onto cubic UiO-66(Zr)–NH_2_ NPs. The heterostructure shows outstanding photocatalytic activity for solar-driven water splitting reactions (HER, OER, and OWS) compared to the individual MOFs. UiO-66(Zr)–NH_2_@MIL-88B(Fe) exhibited hydrogen and oxygen production activity for the OWS with the values of 690 and 279 μmol g^–1^ in 3 h, respectively. Importantly, this photocatalyst exhibits AQY for OWS in the visible region (from 400 to 450 nm) of about 0.9% that compares favorably with previous studies using MOF-based photocatalysts. The high activity of the hybrid MOF-on-MOF heterostructure is proposed to derive from the increased photoinduced charge carrier separation efficiency that operates *via* a Z-scheme mechanism. This mechanism was evidenced by several characterization techniques as the selective photodeposition of Pt and PbOx or CoOx NPs, respectively, on the reductive or oxidative sites of the heterostructure, as well as transient photocurrent measurements, and *in situ* XPS, EIS, TAS, and TRPL analyses.

We consider that this study exemplifies the potential of hybrid MOF-on-MOF architectures based on the rational epitaxial growth of individual MOFs favoring the epitaxial contact interface and ensuing the challenge of solar-driven photocatalytic OWS.

## Experimental and Methods Section

### Synthetic Procedures

#### Synthesis of UiO-66(Zr)–NH_2_ NPs

The synthesis was adapted from the literature.^34^ 2-Aminoterephthalic acid (110 mg; 0.6 mmol) was dissolved in 140 mL of DMF in a round-bottom flask, and 150 μL of triethylamine was added with a pipette. The mixture was stirred for 10 min at 600 rpm. Acetic acid (21 mL; 0.367 mol) was then poured into the flask, and the solution was stirred 10 min more. Finally, a 10 mL DMF solution of zirconyl chloride octahydrate (193.7 mg; 0.6 mmol) was added to the mixture with stirring for 5 min. The mixture was transferred into a 250 mL jar briefly sonicated, which was placed into a preheated oven at 120 °C for 6 h. At the end of the reaction, the jar was removed from the oven and allowed to cool to room temperature. The yellow solid was recovered by centrifugation (5′, 8000 rpm) and washed three times with DMF. For the synthesis of hybrid MOFs, the NPs were dried with vacuum at room temperature, overnight. For characterization, the NPs were washed twice with DMF and twice with MeOH and collected by centrifugation (5′, 8000 rpm). The air-dried solid was placed in a Soxhlet apparatus and washed in MeOH overnight. The powder was finally dried under a vacuum at 80 °C overnight.

#### Synthesis of MIL-88B(Fe)

The reaction conditions were modified from the literature.^35^ Terephthalic acid (271.2 mg; 1.6 mmol) was dissolved in 6 mL of DMF with the addition of 180 μL of triethylamine. Then, a DMF solution of 2 mL of iron chloride hexahydrate (441.2 mg; 1.6 mmol) was added slowly, and the vial was briefly sonicated. The latter was placed in an oven and heated at 100 °C for 12 h. After cooling to room temperature, the resulting orange solid was recovered by centrifugation and washed twice with DMF and twice with MeOH (5′, 8000 rpm). The air-dried solid was placed in a Soxhlet and washed in MeOH overnight. The powder was finally dried under vacuum at 80 °C overnight.

#### Synthesis of UiO-66(Zr)–NH_2_@MIL-88B(Fe)

The reaction conditions were modified from the literature,^35^ and the hybrid was produced in batches of six reactions. In a vial, 16.8 mg of the vacuum-dried UiO-66-NH_2_ NPs were redispersed in 3 mL of DMF, and 47.7 mg (0.281 mmol) of terephthalic acid was added. The mixture was sonicated for 30 min and then transferred to a vial with a Teflon cap. To this mixture were added a DMF solution of 3 mL of iron chloride hexahydrate (77.5 mg; 0.281 mmol) and 32 μL of triethylamine. The vial was briefly sonicated, placed in an oven and heated to 100 °C for 12 h. After cooling to room temperature, the resulting orange solid was recovered by centrifugation and washed twice with DMF, and twice with MeOH (5′, 8000 rpm). The air-dried solid was placed in a Soxhlet and washed in MeOH overnight. The powder was finally dried under vacuum at 80 °C overnight.

### Characterization Techniques

For SEM, a drop of the sample dispersed in EtOH was deposited on a Si substrate, and the images were recorded on a SCIOS 2 FESEM with focused iron beam (FIB). TEM samples were dispersed in EtOH, and 10 μL of the mixture was dropped on a carbon/copper 200 mesh grid from TED PELLA and dried in air. The images were observed on a HITACHI HT7800 HRTEM with a 20 Mpx CMOS EMSIS XAROSA digital camera. ImageJ software was used to measure UiO-66(Zr)–NH_2_ NPs. EDX mapping was performed on a high-resolution transmission electron microscope TECNAI equipped with a CCD GATAN camera. STEM–EDX images were acquired on a field-emission TEM (JEOL JEM-2100F instrument) operating at 200 kW and coupled with an EDX detector. ^1^H NMR data were collected with Bruker Avance III (300 MHz) equipment. Few mg of the samples were previously digested in ammonium fluoride deuterium oxide solution (2M) and heated at 50 °C for 2 days to ensure complete digestion. The metal content was determined after sample digestion by ICP–MS with an Agilent 7900 ICP–MS quadrupole. Leaching of metals from the MOF to the reaction media was determined by ICP–OES after the reaction. FT-IR measurements were performed on an ALPHA II spectrometer (Bruker) in the range of 350–4000 cm^–1^ using the ATR accessory with a diamond window; solid samples were dried in an oven at 100 °C overnight to remove physisorbed water. TGA profiles were collected using a TGA 550 (TA Instruments) at temperatures from 25 to 700 °C in air with a heating rate of 10 °C min^–1^. XRPD patterns were obtained using an X-ray diffractometer (PANalytical Empyrean) with copper as a radiation source (Cu–Kα 1.5418 Å) operating at 40 mA and 45 kV and equipped with an X’Celerator detector. Measurements were collected on a high-throughput screening (HTS) platform or with capillaries. For flexibility measurements, capillaries of the samples activated at 80 °C under vacuum overnight were soaked in each solvent overnight and measured directly with the diffractometer. N_2_ and CO_2_ isotherms were measured with a TRISTAR-2 apparatus (Micromeritics), respectively, at 77 and 273 K. Before the measurements, samples were activated at 150 °C under vacuum overnight. Surface area was calculated by using the Brunauer–Emmett–Teller (BET) equation from the adsorption curve. XPS of solid samples was carried out employing a SPECS spectrometer equipped with an MCD-9 detector using a monochromatic Al (Kα = 1486.6 eV) X-ray source, calibrating the binding energy with a C 1s peak at 284.4 eV as a reference. The software employed for the spectra deconvolution was CASA software. The energy values of the HOCO of the solids versus the Fermi level (*E*_υ_^f^) were estimated by means of XPS. The HOCO position with respect to NHE (*E*_υ_^NHE^) was calculated from the equation *E*_υ_^NHE^ = *E*_υ_^f^ + ϕ_sp_ + *E*_0_^SHE^, where ϕ_sp_ is the work function of the spectrometer used for the measurements (4.244 eV) and *E*_0_^SHE^ is the energy of the SHE with respect to the vacuum level of the electron with the value of −4.44 eV. An *in situ* XPS experiment was carried out measuring the sample UiO-66(Zr)–NH_2_@MIL-88B(Fe) before and after 20 min irradiations with an optical fiber connected to a Hg–Xe lamp (150 W). Diffuse reflectance UV–Vis spectra of solid samples were recorded using a Varian spectrometer model Cary 5000. UV–Vis spectroscopy was employed to estimate the optical band gaps by representing the Tauc plot, and the conduction band energy minimum of each material solid was obtained by the difference between the *E*_υ_^NHE^ value and optical band gap.

### Photocatalytic Experiments

All photocatalytic experiments were performed at least in duplicate trials. Presented photocatalytic data correspond to the average of the different measurements carried out with the corresponding deviation error bar.

#### Photocatalytic OWS

MOF suspension was prepared in a quartz reactor (51 mL) containing 5 mg of MOF photocatalyst and 10 mL of Mili-Q water. As a control experiment, a physical mixture of UiO-66(Zr)–NH_2_ and MIL-88B(Fe), having similar proportions of the individual MOFs in the heterostructure, was prepared. Then, the reactor was subjected to sonication at 450 W for 15 min to obtain a good dispersion and later purged with argon during 1 h to evacuate air from inside. The suspended MOF under stirring was irradiated with a commercially available Hg–Xe lamp (150 W, Hamamatsu ref L8253; Hamamatsu spotlight source L9566-04 and light guide A10014-50-0110) with or without a commercially available AM 1.5G type filter (Lasing ref 81094) to obtain simulated sunlight irradiation. In some cases, commercially available transmittance filters (Thorlabs, ref FGUV11-UV, FELH0400) were also used to study the influence of radiation intensity on the photocatalytic activity. The measured irradiance when using the full spectrum of Hg–Xe lamp was 480 mW/cm^2^, and this value decreased in the presence of an AM 1.5G, a FGUV11-UV, or a FELH0400 filter to 262, 18.55, and 411 mW/cm^2^, respectively. In another experiment, commercially available bandpass filters at 400, 450, 500, and 550 nm (Thorlabs, ref FBH400-10, FBH450-10, FBH500-10, FBH550-10) were employed to estimate the AQY but using UiO-66(Zr)–NH_2_@MIL-88B(Fe) during the photocatalytic OWS. Gaseous products of interest from these experiments were analyzed by connecting through the head space directly to the reactor with an Agilent 490 Micro GC system (Molsieve 5 Å column using Ar as the carrier gas) without manual handling. During the experiment, the temperature was monitored, and the pressure was controlled by the manometer adapted to the photoreactor. The stability of UiO-66(Zr)–NH_2_@MIL-88B(Fe) was studied after the photocatalytic OWS by ICP-OES and XRPD.

#### Photocatalytic Hydrogen or Oxygen Evolution Reactions

For HER, a MOF suspension (5 mg) was prepared in a quartz reactor (51 mL) with a mixture of Mili-Q water (8 mL) and MeOH (2 mL) as the electron donor. As a control experiment, a physical mixture of UiO-66(Zr)–NH_2_ and MIL-88B(Fe), having similar proportions of the individual MOFs in the heterostructure [57/43 mass ratio of MIL-88B(Fe) and UiO-66(Zr)–NH_2_, respectively], was prepared. To study the influence of the electron donor, HER was carried out with different quantities of MeOH (0.6 and 0.2 mL) and by adding Mili-Q water up to a total volume of 10 mL. In OER, a MOF suspension (5 mg) was prepared in a quartz reactor (51 mL) containing Mili-Q water (10 mL) and sodium persulfate (700 mg) as the electron quencher. To study the influence of the electron quencher, the reaction of the OER was carried out with different quantities of sodium persulfate (300 and 50 mg). Each reactor was subjected to sonication at 450 W for 15 min to obtain a uniform dispersion. After that, the reactors were purged with argon during 1 h to evacuate air from inside. The suspended MOF under stirring was irradiated with a commercially available Hg–Xe lamp (150 W, Hamamatsu ref L8253; Hamamatsu spotlight source L9566-04 and light guide A10014-50-0110) with a commercially available AM 1.5G type filter (Lasing ref 81094) to obtain simulated sunlight irradiation. The irradiance measured using the UV–vis Hg–Xe lamp was 480 mW/cm^2^, and it was 262 mW/cm^2^ in the presence of AM 1.5G. In another experiment, commercially available bandpass filters at 400, 450, 500, and 550 nm (Thorlabs, ref FBH400-10, FBH450-10, FBH500-10, and FBH550-10) were employed to estimate the AQY but using UiO-66(Zr)–NH_2_@MIL-88B(Fe) during the photocatalytic HER. The reaction evolution was followed by an Agilent 490 Micro GC system (Molsieve 5 Å column using Ar as the carrier gas) without manual handling. During the experiment, the temperature was monitored, and the pressure was controlled by the manometer adapted to the photoreactor. The stability of UiO-66(Zr)–NH_2_@MIL-88B(Fe) was studied after the photocatalytic HER by techniques different from ICP-OES or XRPD.

#### Heterostructure Stability in Water and Water/MeOH

The stability of the UiO-66(Zr)–NH_2_@MIL-88B sample in water and the H_2_O/MeOH (8:2) mixture was studied by preparing a suspension containing 5 mg of MOF photocatalyst and 10 mL of volume. The suspended MOF was stirred for 3 h. The solid was filtered off, and the reaction liquid was subjected to ICP-OES to analyze the possible metal leaching.

### Photophysical Measurements

#### Time-Resolved Photoluminescence Spectroscopy Measurements

An EasyLife X lifetime fluorescence spectrometer was used for TRPL spectroscopy measurements. 1 mg/mL suspensions of each MOF were prepared in ACN and sonicated for 20 min (450 W sonicator). Then, more diluted suspensions were prepared until we reached 0.3 UV–vis absorbance at 350 nm. Before measurement of TRPL, the cuvettes were purged with argon for 10 min to remove dissolved oxygen. Equipment lifetime limitation was measured with a cuvette filled with an ACN solvent.

#### Laser Flash Photolysis Measurements

Suspensions of each MOF were prepared in ACN at 1 mg mL^–1^, and then the concentrations were adjusted to obtain a 0.3 optical density at 355 nm for analysis using a Q switched Nd/YAG laser (Quantel Brilliant, 10 mJ/pulse, 5 ns fwhm) excited with the 355 and 266 nm harmonic and coupled to a mLFP-122 Luzchem miniaturized detection equipment. This transient absorption spectrophotometer consists of a 300 W ceramic xenon lamp, 125 mm monochromator, Tektronix TDS-2001C digitizer, compact photomultiplier and power supply, liquid cell holder, fiber-optic connectors, and computer interfaces. The software package has been developed in the LabVIEW environment from National Instruments. The laser flash photolysis equipment supplies 5 V trigger pulses with a programmable frequency and delay. The rise time of the detector/digitizer is ∼3 ns up to 300 MHz (2.5 GHz sampling). The monitoring beam is provided by a ceramic Xe lamp through fiber-optic cables. The laser pulse is probed by a fiber that synchronizes the LFP system with the digitizer operating in the pretrigger mode. Finally, transient spectra of the persistent suspensions were recorded using 10 × 10 mm quartz cells and were bubbled for 15 min with Ar before data acquisition. Each decay or data point corresponds to the average of five signals to increase the signal-to-noise ratio.

### Photocurrent Experiments

For photocurrent experiments, a standard three-electrode electrochemical cell was employed. A thin layer of MOF-based materials was deposited on carbon glass and employed as a working electrode. A platinum wire was used as a counter electrode and a standard calomel electrode (SCE) as the reference electrode. Before the measurements, the cell system was purged with Ar through the electrolyte (tetra-*n*-butylammonium hexafluorophosphate, TBAPF_6_) ACN solution for 20 min to remove the oxygen from inside. The current was measured under dark or illumination conditions by polarizing the working electrode at potentials from 1.4 to 0.3 V. The illumination of the working electrode was performed by using an optical fiber connected to a Hg–Xe lamp (150 W) with an AM 1.5G filter.

### Photodeposition Method

Photodepositions were carried out in a fluorimeter quartz cuvette (Friedrich & Dimmock) by preparing a 4 mL water solution containing 5 mg of H_2_PtCl_6_ (8 wt % of Pt in H_2_O) (CAS 16941-12-1) with either 3 mg of Pb(NO_3_)_2_ of 99% purity (CAS 10099-74-8) or 2 mg Co_3_O_4_ of ≥99% purity (CAS 1307-96-6). To each solution was added 3 mg of UiO-66(Zr)–NH_2_@MIL-88B(Fe). Then, both suspensions were irradiated using an optic fiber with a 150 W Hg–Xe lamp in the presence of Argon flow (25 mL/min) during 3h. Finally, solids were recovered by filtration, washed with Mili-Q water, and supported on a TEM grid for analysis.

## Data Availability

Thibaut Le Huec, Antón López-Francés, Isabel Abánades Lázaro, Sergio Navalón, Herme G. Baldoví Mónica Giménez-Marqués, Heteroepitaxial MOF-on-MOF photocatalyst for solar-driven water splitting, ChemRxiv. 2024; doi:10.26434/chemrxiv-2024-k0z70 (accessed July 8, 2024).

## Supporting Information Available

Materials and reagents; TEM image and size distribution; XRPD analysis; pore size distribution analysis; TGA; MOF/residue ratios; ^1^H NMR spectrum; mass ratio calculations from ICP–MS and NMR analysis; ATR-FTIR spectra; solvent-dependent XRPD studies; surface area plots for BET calculations; CO_2_ sorption isotherms; XPS spectra; Tauc plots; photocatalytic activity upon different irradiation conditions; H_2_^18^O mass spectrum; SEM images and elemental mapping after five cycles of OWS; TGA profiles of UiO-66(Zr)–NH_2_ before and after exposure to epitaxial conditions; TAS of UiO-66(Zr)–NH_2_@MIL-88B(Fe); TRPL spectra; STEM images for photodeposition studies; XPS prior to and after irradiation; and photocatalytic HER and OER upon different MeOH and Na_2_S_2_O_8_ amounts (PDF)
