# Supplementary material for: Heteroepitaxial MOF-on-MOF Photocatalyst for Solar-Driven Water Splitting
Source: ACS Nano. 2024 Jul 30;18(31):20201–12. doi: 10.1021/acsnano.4c03442 (PMC11308772; doi:10.1021/acsnano.4c03442)
Supplement: Supplementary file 1 — nn4c03442_si_001.pdf [file nn4c03442_si_001.pdf]

Supporting Information

Heteroepitaxial MOF-on-MOF photocatalyst for solar-driven water splitting

Thibaut Le Huec,[a],$ Antón López-Francés,[b],$ Isabel Abánades Lázaro,[a] Sergio Navalón, [b] Herme G. Baldoví, [b], and Mónica Giménez-Marqués[a],**

[a] Thibaut Le Huec, Isabel Abánades Lázaro and Mónica Giménez-Marqués

Instituto de Ciencia Molecular (ICMol), Universidad de Valencia,

C/ Catedrático José Beltrán Martínez, 2, 46980 Paterna, Valencia, Spain

[b] Antón López-Francés, Sergio Navalón, Herme G. Baldoví

Departamento de Química, Universitat Politècnica de València,

C/Camino de Vera, s/n, 46022, Valencia, Spain

■ Experimental Section

■ Supplementary Figures and tables

Figure S1. TEM analysis and size distribution

Figure S2. X Ray Powder Diffraction (XRPD) of UiO-66(Zr)-NH_2

Figure S3. Pore size distribution analysis

Figure S4. Thermogravimetric analysis

Table S1. Table with experimental and theoretical TGA MOF/Residue ratios

Figure S5. ^1H NMR spectrum of UiO-66(Zr)-NH_2@MIL-88B(Fe)

Table S2. Mass ratio calculations obtained by ICP-MS and NMR analysis

Figure S6. ATR-FTIR spectra

Figure S7. Solvent-dependent XRPD measurements

Figure S8. Surface area determination by Brunauer–Emmett–Teller (BET) theory.

Figure S9. CO_2 sorption isotherms

Figure S10-13. High resolution X-ray photoelectron spectroscopy (XPS)

Figures S14 UV-Vis DRS diagram

Figure S15 Tauc plots

Figure S16. Photocatalytic activity for OWS upon different irradiation conditions

Figure S17. Mass spectrum after OWS reaction

Figure S18. SEM images and elemental mapping after cycles of OWS

Figure S19. TGA profile of UiO-66(Zr)-NH_2 before and after epitaxial growth

Figure S20-22. TAS of UiO-66(Zr)-NH_2@MIL-88B(Fe)

Figure S23. TRPL spectra

Figure S24-29. STEM images for photo deposition studies.

Figure S30. XPS of UiO-66(Zr)-NH_2@MIL-88B(Fe) prior and after irradiation.

Figure S31. Study of sacrificial agents' effect over photocatalytic activity.

■ Experimental Section

Materials and reagents

Zirconium (IV) oxide chloride octahydrate EMSURE®, Iron(III) chloride hexahydrate (98-102%) and also triethylamine (99.5%) were purchased from Sigma-Aldrich. Terephthalic acid (98%), 2-Aminoterephthalic acid (99%) and Fumaric acid (99%) were purchased from Alfa Aesar. Dimethylformamide and Methanol with HPLC grade as well as acetic acid glacial were bought from Scharlau.

■ Supplementary Figures and tables

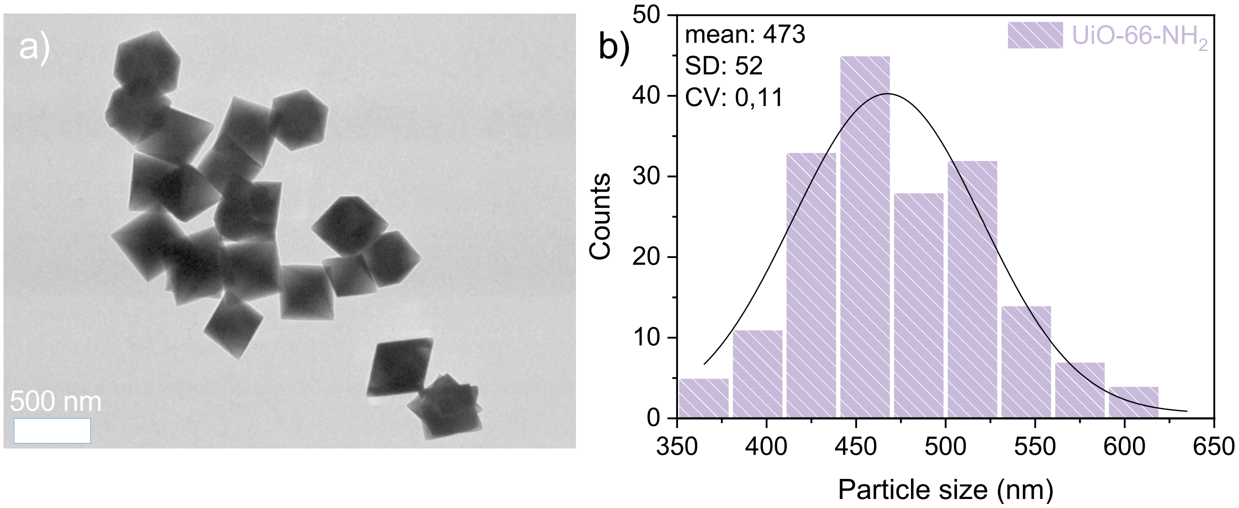

Figure S1. a) TEM image and b) distribution size counts of UiO-66-NH_2 NPs (based on 3 different batches of 60 nanoparticles).

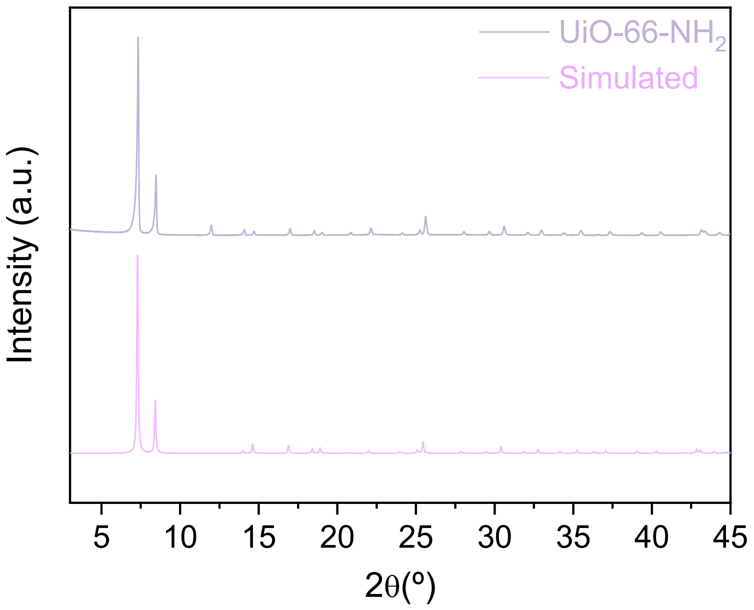

Figure S2. XRPD experimental pattern of UiO-66(Zr)-NH_2 and the corresponding simulated pattern corresponding to UiO-66(Zr)-NH_2 from single crystal resolution.

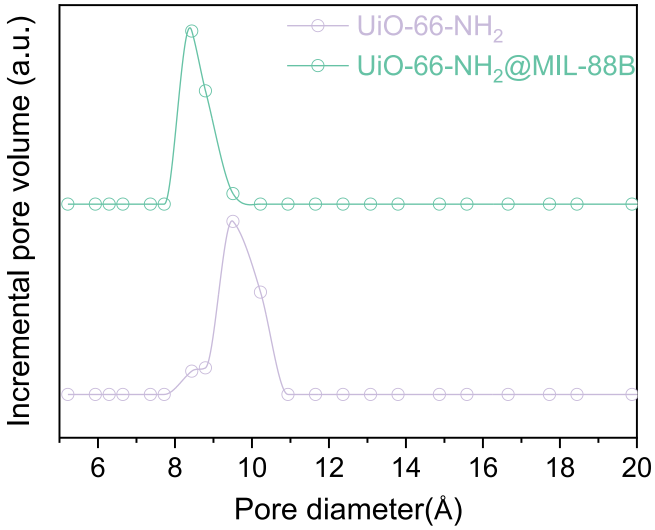

Figure S3. a) Pore size distribution measurements (NLDFT model for cylindrical pores) of UiO-66(Zr)-NH_2 and UiO-66(Zr)-NH_2@MIL-88B(Fe).

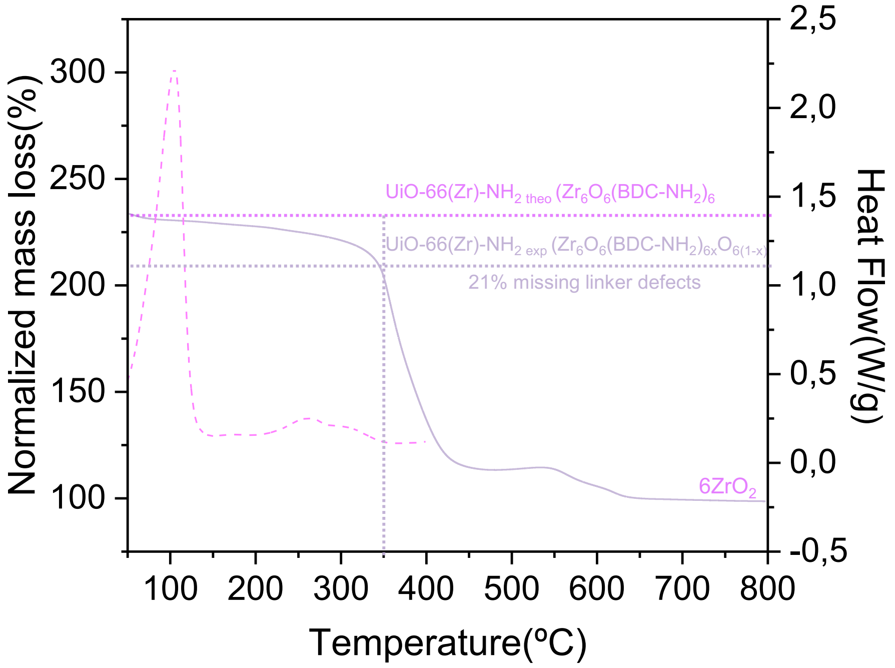

Figure S4. Thermogravimetric analysis (purple line) and differential scanning calorimetry (pink dash line) of experimental UiO-66(Zr)-NH_2. x represents the number of linkers per zirconium in the sample.

Table S1. Ratios MOF/Residue of UiO-66(Zr)-NH_2 theoretical versus experimental with the decomposition profile normalized to 100 % of the residue. Between 200 and 350 ºC, UiO-66(Zr)-NH_2 is dehydroxylated and the modulators that are still attached to the structure are decomposed.

	Ratio MOF/Residue
$Zr_6O_6(BDC\text{-}NH_2)_6$ (350 °C) (theoretical)	2.32
$Zr_6O_6(BDC\text{-}NH_2)_{6-x}$ (350 °C) (experimental)	2.05

<u>**Calculation of missing linkers defects from TGA:**</u>

The number of linker x per zirconium was estimated through the equations reported in the literature.[3]

$$x = \frac{\text{Rexp } (350°C)^* M_w[ZrO_2] - M_w[ZrO] - M_w[O]}{M_w[BDC\text{-}NH_2] - M_w[O]} = 0.79$$

Hence the number of BDC-NH_2 ligands per unit cell is 6*0.79= 4.76 ligands (6 ligands theoretically per unit cell).

We can also deduce that the percentage of ligand defects is 21 %, being [(1-x)/1]*100

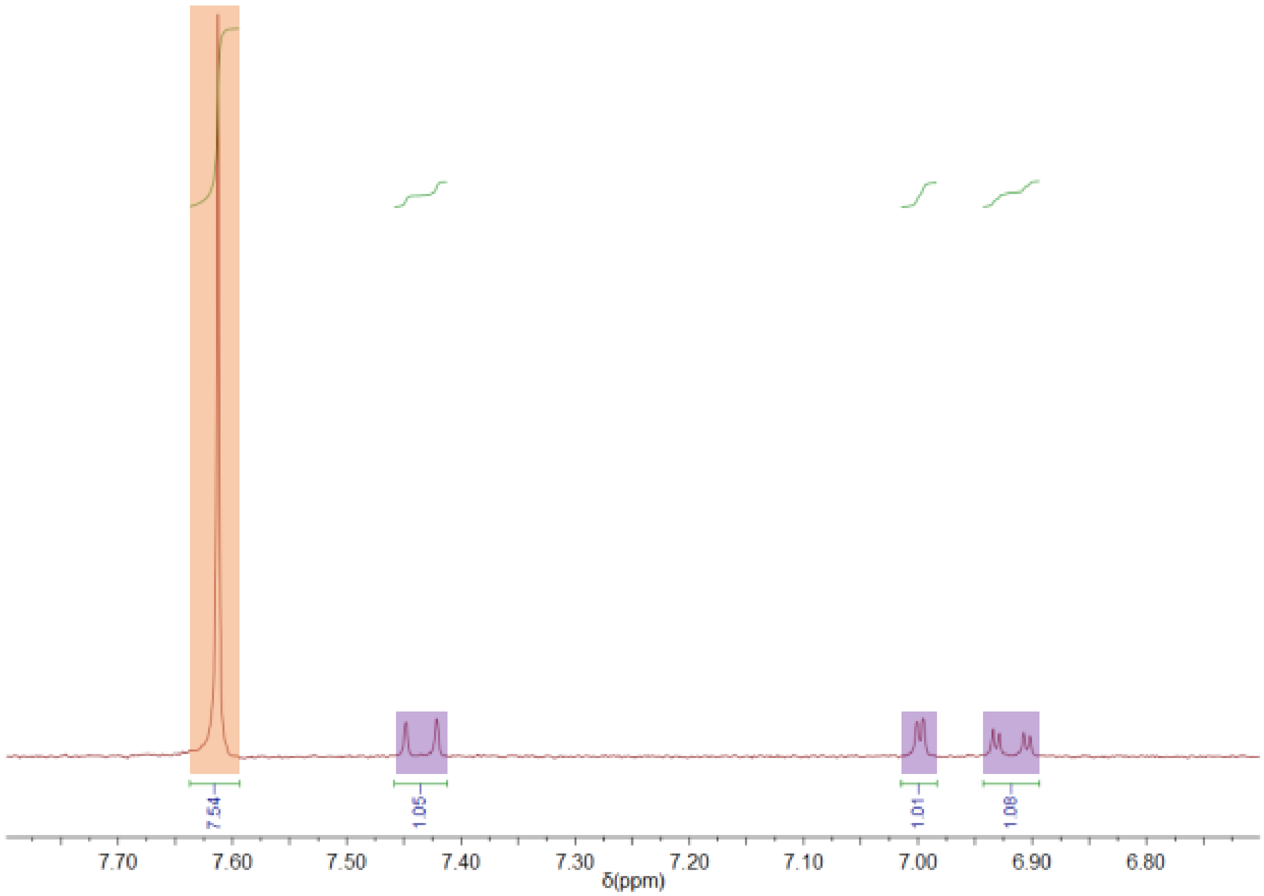

Figure S5. ^{1}H NMR spectrum of UiO-66(Zr)-NH_2@MIL-88B(Fe). The orange and purple bars represent respectively the peaks of terephthalic (a singlet counting for 4 protons, MIL-88B(Fe)) and 2-aminoterephthalic acid (2 doublets and a doublet of doublet counting each for 1 proton, UiO-66(Zr)-NH_2).

Table S2. Mass ratios of MIL-88B(Fe) and UiO-66(Zr)-NH_2 obtained by ICP-MS and NMR analysis (calculated from an average of 7 batches). The results are displayed before and after considering the missing linkers defects in UiO-66(Zr)-NH_2.

			Considering missing linker calculated from TGA	
Mass ratio	MIL-88B(Fe)	UiO-66(Zr)-NH_2	MIL-88B(Fe)	UiO-66(Zr)-NH_2
ICP-MS	54.1	45.9	56.4	43.6
NMR	61.2	38.8	57.8	42.2

<u>**Calculation of MOF mass ratios from NMR:**</u>

Thanks to the integration of peaks in NMR we can deduce the molar ratio of BDC (MIL-88B (Fe)) and BDC-NH_2 (UiO-66(Zr)-NH_2) ligands.

$$R_{BDC\text{-}NH_2}\ (\%)=\frac{\dfrac{I_{BDC\text{-}NH_2}}{n_{H^+BDC\text{-}NH_2}}}{\dfrac{I_{BDC\text{-}NH_2}}{n_{H^+BDC\text{-}NH_2}}+\dfrac{I_{BDC}}{n_{H^+BDC}}}\times 100$$

$$R_{BDC}\ (\%)=100-R_{BDC\text{-}NH_2}$$

Where I_{BDC} and $I_{BDC\text{-}NH_2}$ are the integration values corresponding to the peaks of BDC and BDC-NH_2 ligands. Furthermore, the number of protons for each ligand peak are expressed as n_{H^+BDC} and $n_{H^+BDC\text{-}NH_2}$ (BDC has 1 peak counting for 4 protons whereas BDC-NH_2 3 peaks counting each for 1 proton).

Hence, we can obtain the UiO-66-NH_2 and MIL-88B molar ratio:

$$R_{UiO\text{-}66\text{-}NH_2}(\%)=\frac{\dfrac{R_{BDC\text{-}NH_2}}{n_{BDC\text{-}NH_2}}}{\dfrac{R_{BDC\text{-}NH_2}}{n_{BDC\text{-}NH_2}}+\dfrac{R_{BDC}}{n_{BDC}}}\times 100$$

$$R_{MIL\text{-}88B}\,(\%) = 100 - R_{UiO\text{-}66\text{-}NH_2}$$

With n_{BDC} and $n_{BDC\text{-}NH_2}$ respectively the number of ligands in one unit cell of MIL-88B(Fe) and UiO-66(Zr)-NH_2 (i.e. UiO-66(Zr)-NH_2 and MIL-88B(Fe) have respectively 6 BDC-NH_2 and 3 BDC ligands in their unit cell)

In presence of "missing linkers" in UiO-66(Zr)-NH_2, $n_{BDC\text{-}NH_2}$ will be modified.

(Example: In our case, 4.76 2-aminoterephthalic ligands per Zr_6 unit).

Finally, we can calculate the UiO-66-NH_2 mass ratio:

$$M_{UiO\text{-}66\text{-}NH_2}(\%) = \frac{\dfrac{R_{BDC\text{-}NH_2}}{M_w UiO\text{-}66\text{-}NH_2}}{\dfrac{R_{BDC\text{-}NH_2}}{M_w UiO\text{-}66\text{-}NH_2} + \dfrac{R_{BDC}}{M_w MIL\text{-}88B}} \times 100$$

$$M_{MIL\text{-}88B}\,(\%) = 100 - M_{UiO\text{-}66\text{-}NH_2}$$

Where M_wMIL-88B and M_wUiO-66-NH_2 are the corresponding molecular weight of each MOF.

<u>**Calculations of MOF mass ratios from ICP-MS:**</u>

Starting with the mass concentration of Fe and Zr in the sample we can deduce their molar ratio:

$$R_{Zr}(\%) = \frac{\dfrac{C_m\,Zr}{M_w\,Zr}}{\dfrac{C_m\,Zr}{M_w\,Zr} + \dfrac{C_m\,Fe}{M_w\,Fe}} \times 100$$

$$R_{Fe}\,(\%) = 100 - R_{Zr}$$

Where C_m Fe and C_m Zr are the mass concentration of Fe and Zr. The corresponding molecular weights of Fe and Zr are expressed as M_w Fe and M_w Zr.

Hence, we can obtain the UiO-66(Zr)-NH_2 molar ratio within the heterostructure:

$$R_{UiO\text{-}66(Zr)\text{-}NH_2}(\%)=\frac{\frac{R_{Zr}}{n_{Zr}}}{\frac{R_{Zr}}{n_{Zr}}+\frac{R_{Fe}}{n_{Fe}}}\times 100$$

$$R_{MIL\text{-}88B}\ (\%)= 100\text{-}R_{UiO\text{-}66\text{-}NH_2}$$

With n_{Fe} and n_{Zr} respectively the number of metals in one unit cell of MIL-88B(Fe) and UiO-66(Zr)-NH_2 (i.e. UiO-66(Zr)-NH_2 and MIL-88B(Fe) have respectively 6 Zr and 3 Fe in their unit cell). To calculate UiO-66(Zr)-NH_2 mass ratio, we use the same formula as for the NMR measurements.

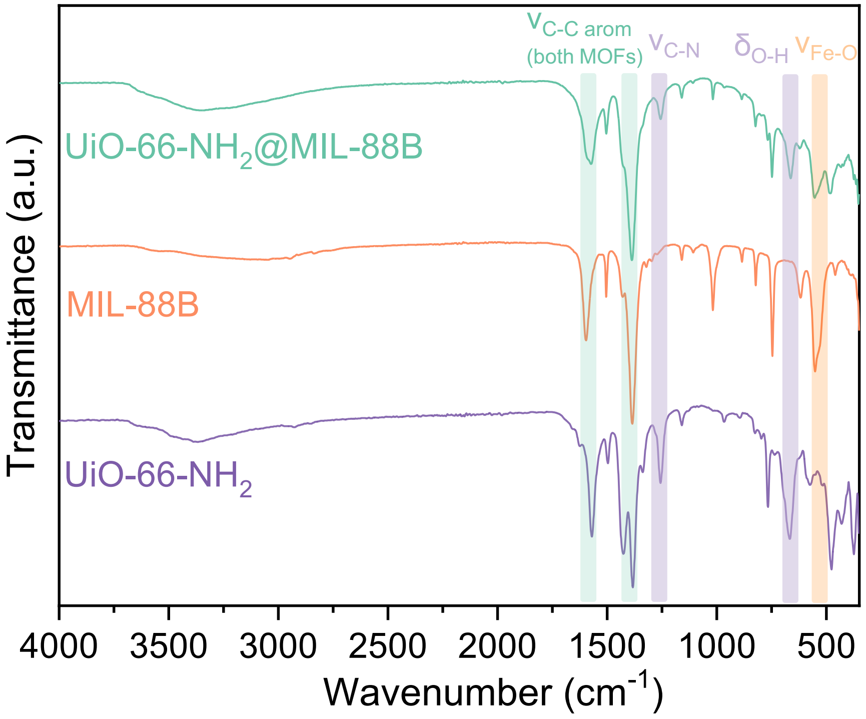

Figure S6. ATR-FTIR spectra of UiO-66(Zr)-NH_2@MIL-88B(Fe) compared to UiO-66(Zr)-NH_2 and MIL-88B(Fe) individual MOFs.

IR spectrum of the hybridized MOF exhibits the combination of the two main COO stretching bands from UiO-66(Zr)-NH_2 and MIL-88B(Fe) in the 1600-1400 cm^{-1} range. The characteristic band at 1500 cm^{-1} corresponding to the C=C aromatic stretching in UiO-66(Zr)-NH_2 or MIL-88B(Fe) MOF is also present. The metallic bands of UiO-66(Zr)-NH_2 at 750 cm^{-1} (stretching Zr-O) and MIL-88B(Fe) at 450 cm^{-1} (Fe-O stretching) can be observed. Finally, the presence of a band at 1250 cm^{-1} which can be attributed to the C-N stretching of the amino group was observed in UiO-66(Zr)-NH_2@MIL-88B(Fe).

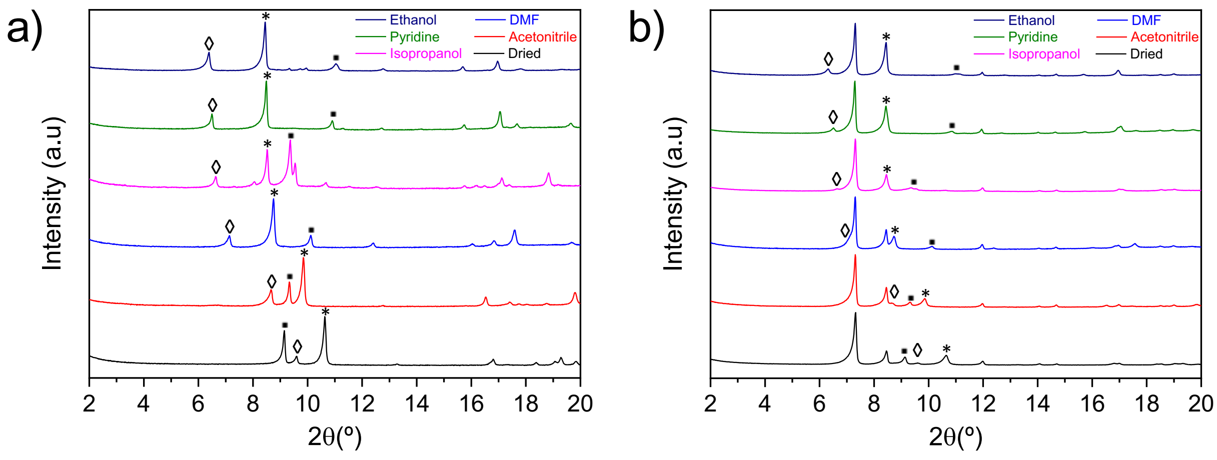

Figure S7. XRPD capillary measurements of a) MIL-88B(Fe) and b) UiO-66(Zr)-NH_2@MIL-88B(Fe) after soaking in different solvents. The rhombus, asterisk and square symbols represent respectively the planes (100), (101) and (002) of MIL-88B(Fe) in both materials.

A set of capillaries containing MIL-88B(Fe) and UiO-66(Zr)-NH_2@MIL-88B(Fe) materials thermally activated to remove physiosorbed solvent from the pores, were soaked in a series of solvents. Upon soaking, the peaks of MIL-88B(Fe) corresponding to (100), (101) and (002) planes shifted as observed on the XRPD pattern, showing a favoured expansion of the material in solvents such as pyridine, ethanol and DMF (open-form) whereas the component tends to shrink in presence of isopropanol or acetonitrile (close-form). A similar solvent-responsive phenomenon was also observed in the hybrid material.

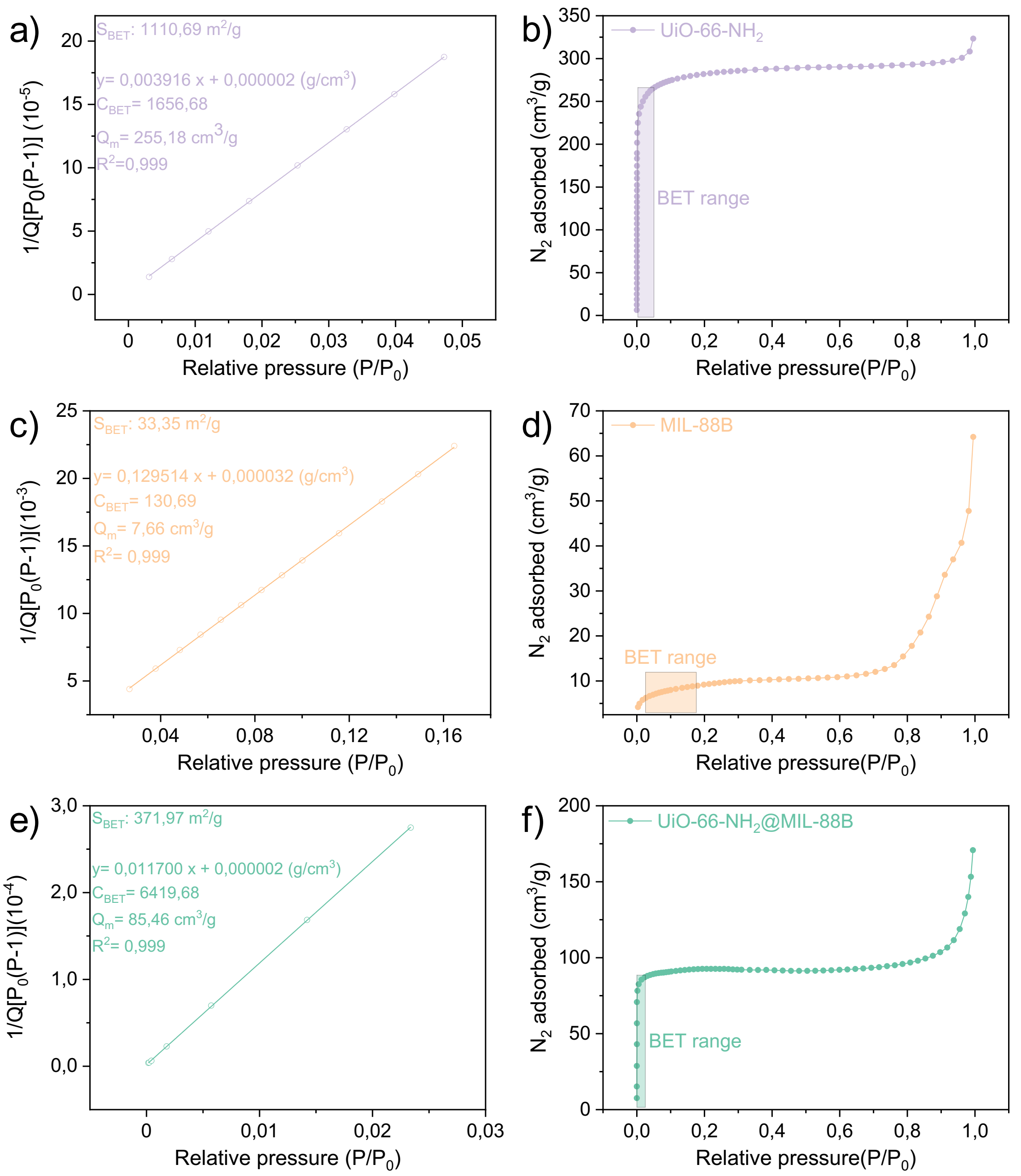

Figure S8. BET surface area plots (a, c, e) and corresponding sorption isotherms (b, d, f) of a) UiO-66(Zr)-NH_2, b) MIL-88B(Fe) and c) UiO-66(Zr)-NH_2@MIL-88B(Fe). The BET calculation range was determined considering the maximum value of the Rouquerol plot and ensuring the fitting of the BET surface linear plot.

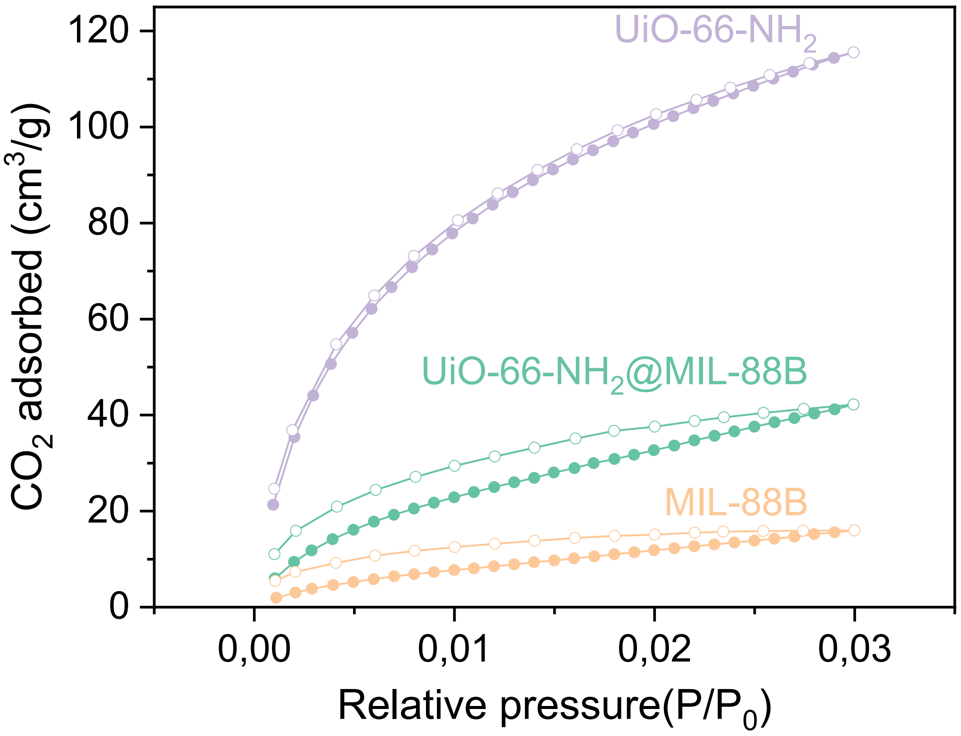

Figure S9. CO_2 sorption (filled spheres) and desorption (empty spheres) isotherms at 273 K of UiO-66(Zr)-NH_2@MIL-88B(Fe) and single UiO-66(Zr)-NH_2 and MIL-88B(Fe) MOFs.

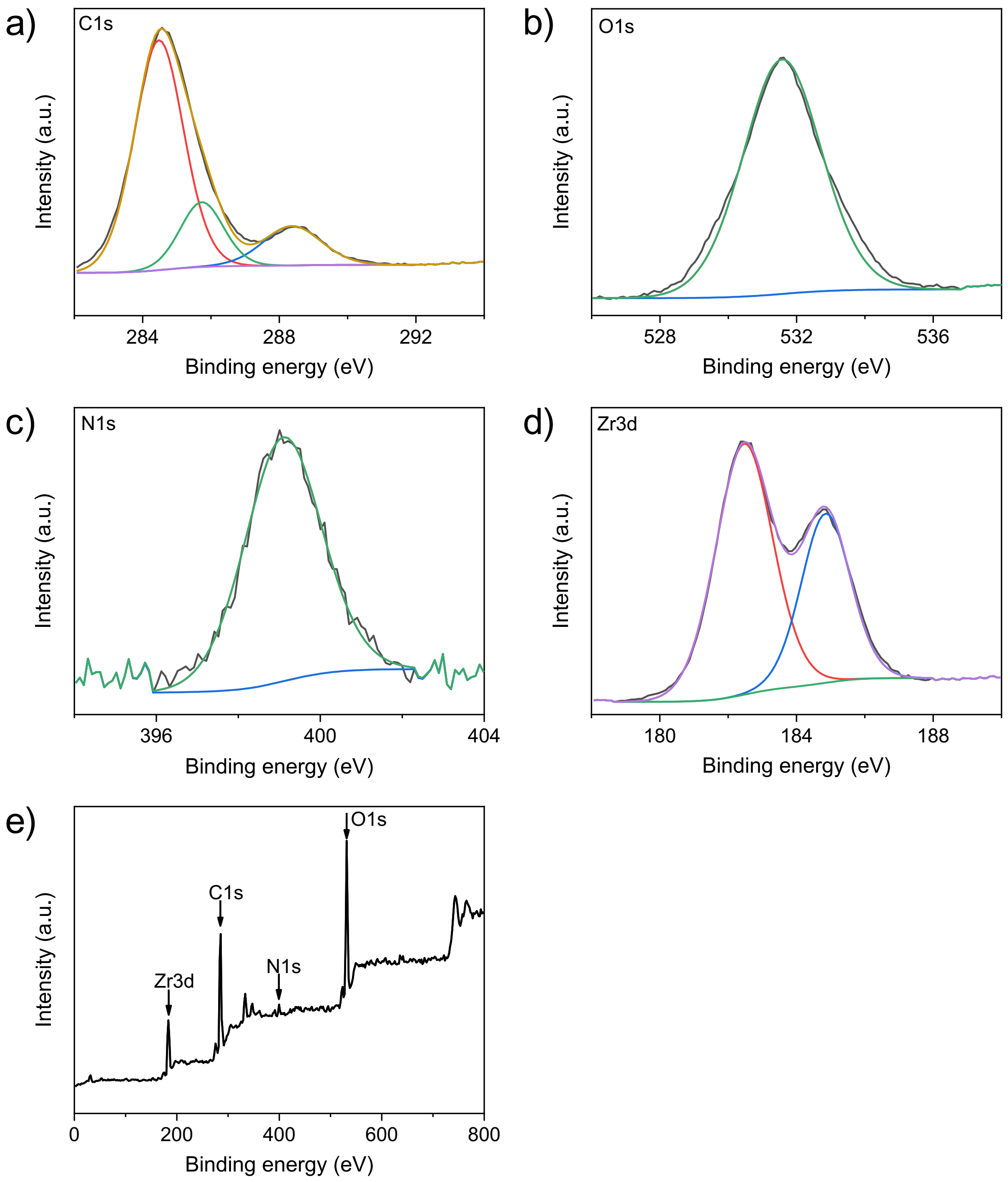

Figure S10. High resolution XPS peaks and the best deconvolution for the C 1s (a), O 1s (b), N 1s(c), Zr 3d (d) and survey scan (e) signals recorded for the UiO-66(Zr)-NH_2.

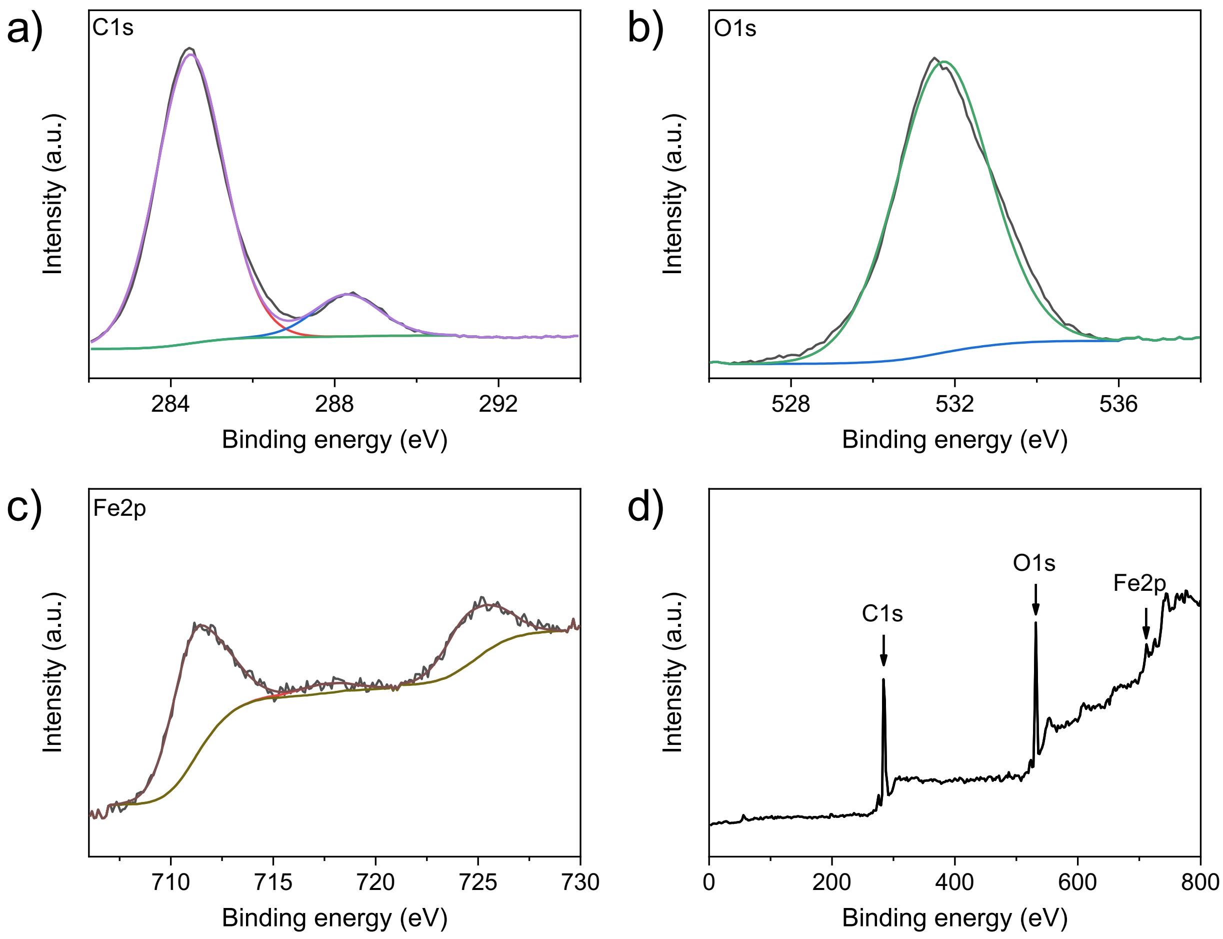

Figure S11. High resolution XPS peaks and the best deconvolution for the C 1s (a), O 1s (b), Fe 2p (c) and survey scan (d) signals recorded for the MIL-88B(Fe).

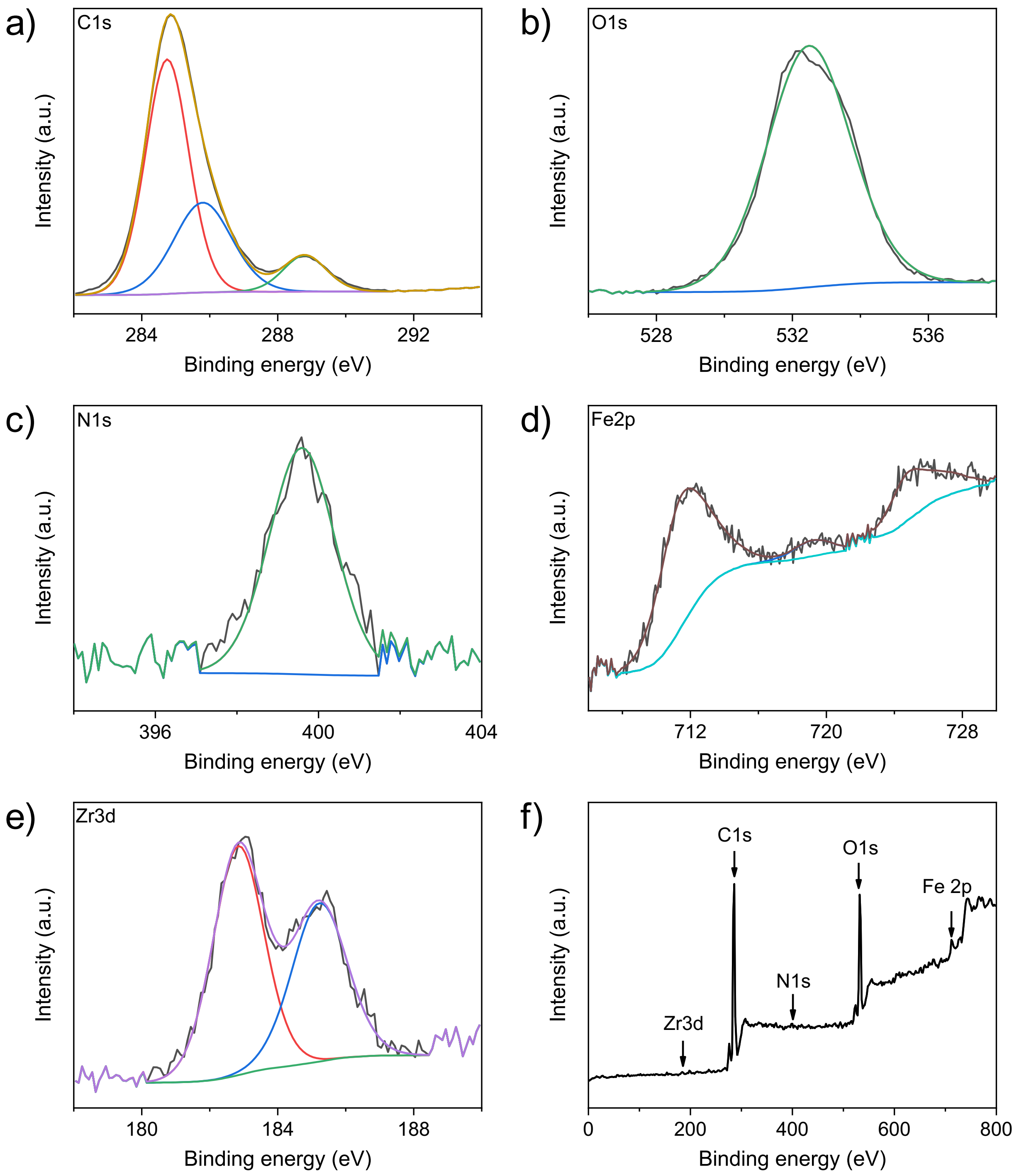

Figure S12. High resolution XPS peaks and the best deconvolution for the C 1s (a), O 1s (b), N 1s(c), Fe 2p (d), Zr 3d (e) and survey scan (f) signals recorded for the UiO-66(Zr)-NH_2@MIL-88B(Fe).

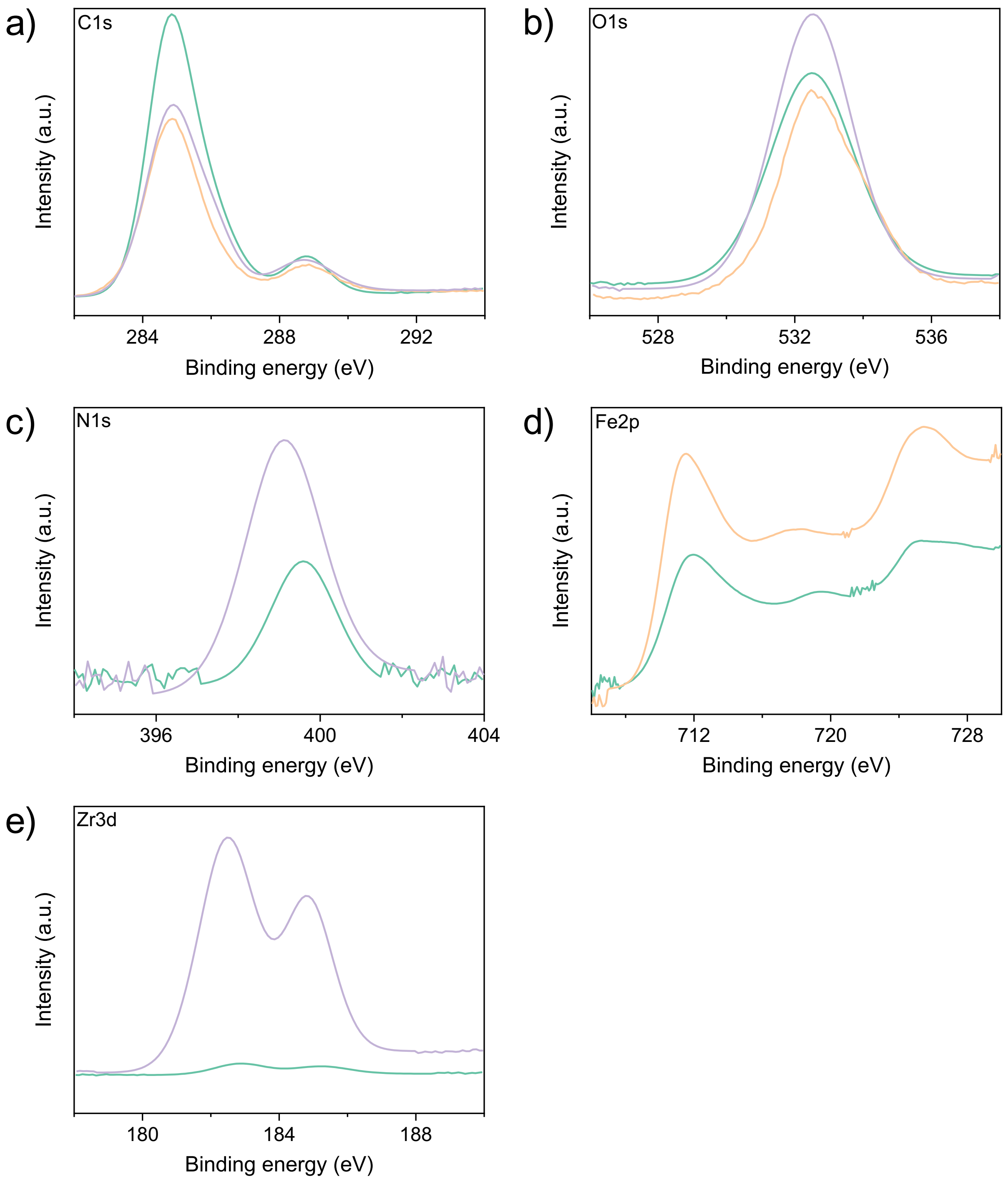

Figure S13. High resolution XPS peaks overlay for the C 1s (a), O 1s (b), N 1s(c), Fe 2p (d) and Zr 3d (e) signals recorded for the UiO-66(Zr)-NH_2 (purple lines), MIL-88B(Fe) (orange lines) and UiO-66(Zr)-NH_2@MIL-88B(Fe) (green lines).

C 1s XPS spectra shows the presence of sp^2 aromatic carbons (284.4 eV) and carboxylate groups (288.0 eV) of the organic ligand. The hybrid heterostructure and the UiO-66(Zr)-NH_2 material that have amino groups also exhibit the corresponding bands in C 1s and N 1s XPS regions at 286.0 and 399.0 eV, respectively. The broad O 1s XPS spectra is associated to the presence of oxygen atoms present in M-O (M: Zr or Fe) and COO– groups at about 529 and 531 eV, respectively. The Zr 3d XPS spectra of the hybrid heterostructure and UiO-66(Zr)-NH_2 materials confirm the presence of Zr(IV) as deduced from the two bands at 182.0 and 184.4 eV corresponding to Zr 3d5/2 and Zr 3d3/2, respectively. XPS Fe 2p of MIL-88B(Fe) and the hybrid heterostructure reveal characteristic features of Fe (III) ions with two bands at 712 and 715 eV associated to Fe 2p3/2 and Fe 2p1/2, respectively.

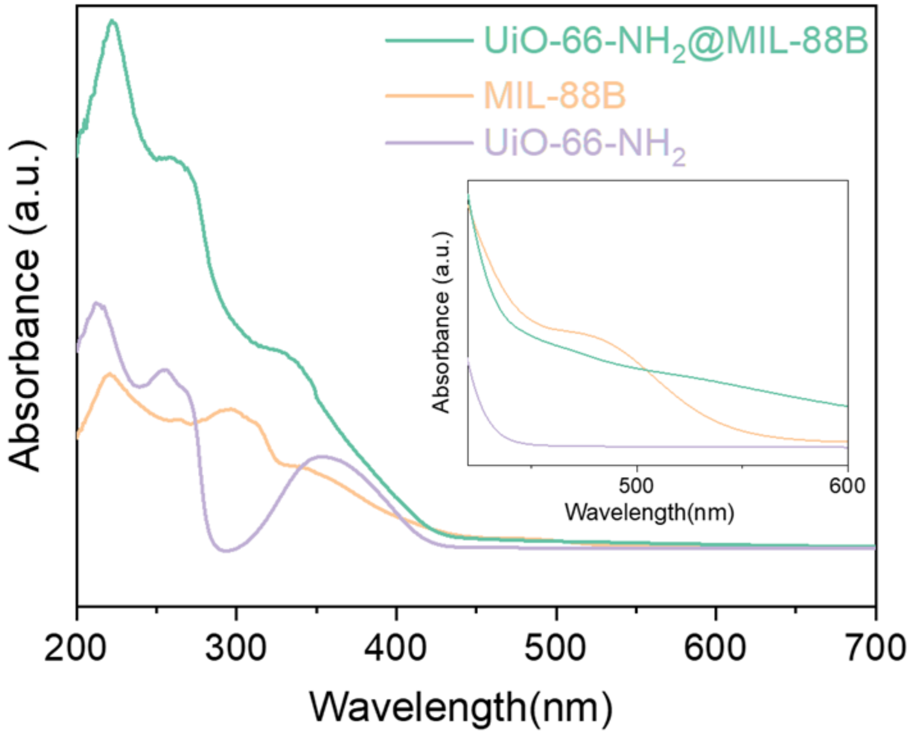

Figure S14. UV-Vis DRS diagram of UiO-66(Zr)-NH_2 (purple), MIL-88B(Fe) (orange) and UiO-66(Zr)-NH_2@MIL-88B(Fe) (green).

The UV-Vis DRS absorption spectrum of UiO-66(Zr)-NH_2 solid is characterized by a band appearing at 260 nm attributed to the presence of the aromatic organic ligand and the Zr-O cluster within the MOF. In addition, the presence of the –NH_2 group on the terephthalate ligand is responsible for the absorption band centered at 340 nm, with a shoulder extending up to about 450 nm. The spectrum of MIL-88B(Fe) also exhibits an absorption of UV light from 200 to 300 nm, mainly associated with the terephthalate organic ligand, with a less intense but characteristic absorption band of the Fe_3-μ-O cluster in the visible region between 450 and 550 nm. The UV-Vis absorption profile of the MOF-on-MOF hybrid material exhibits the

characteristic absorption bands of the counterparts together with a new absorption feature in the visible region from *ca*. 500 to 650 nm, which is associated with the electronically contact created between the two MOF counterparts.

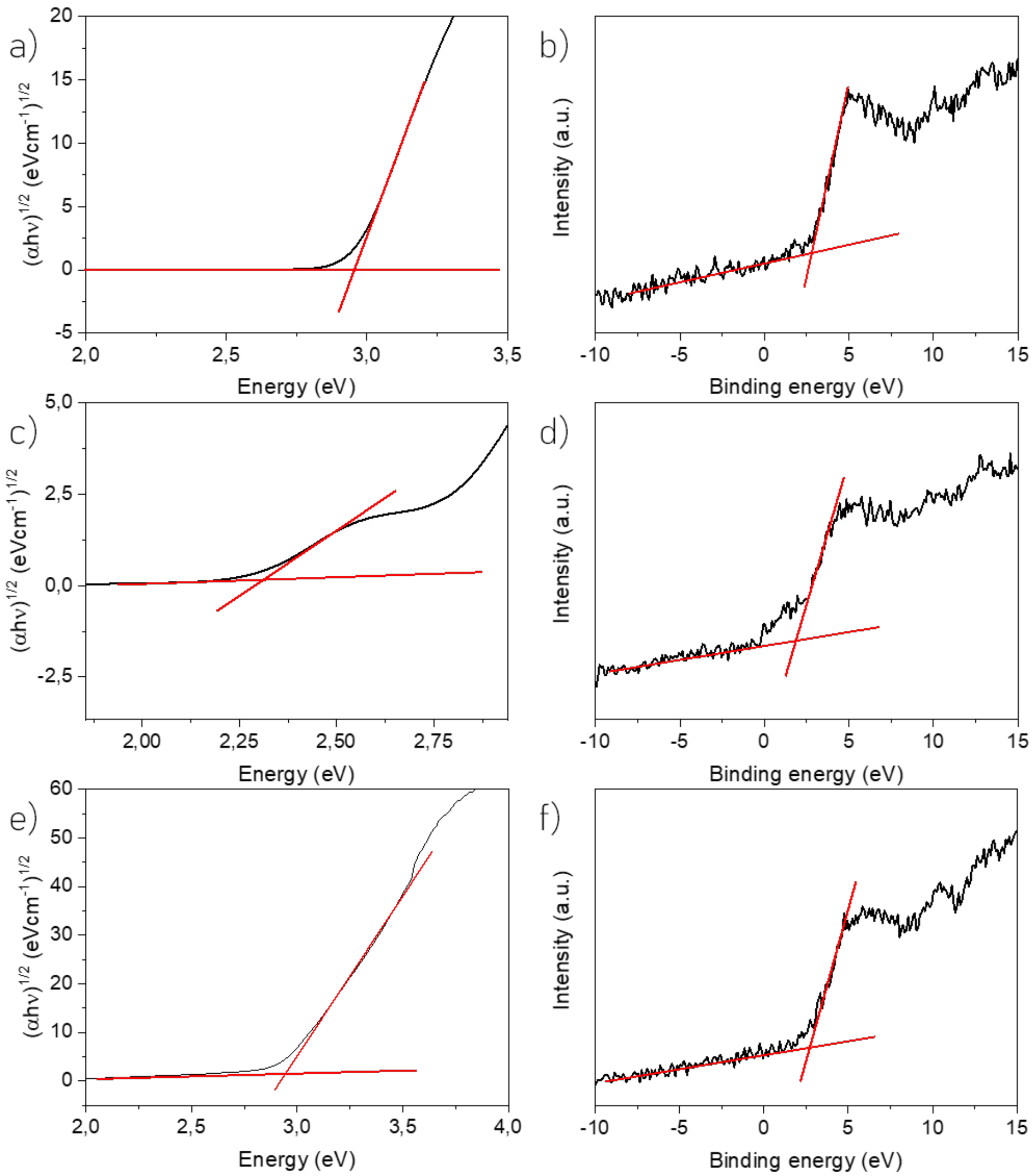

Figure S15. Tauc plot (a, c, e) and XPS analysis of HOCO (b, d, f) for UiO-66(Zr)-NH_2, MIL-88B(Fe) and UiO-66(Zr)-NH_2@MIL-88B(Fe) solids, respectively.

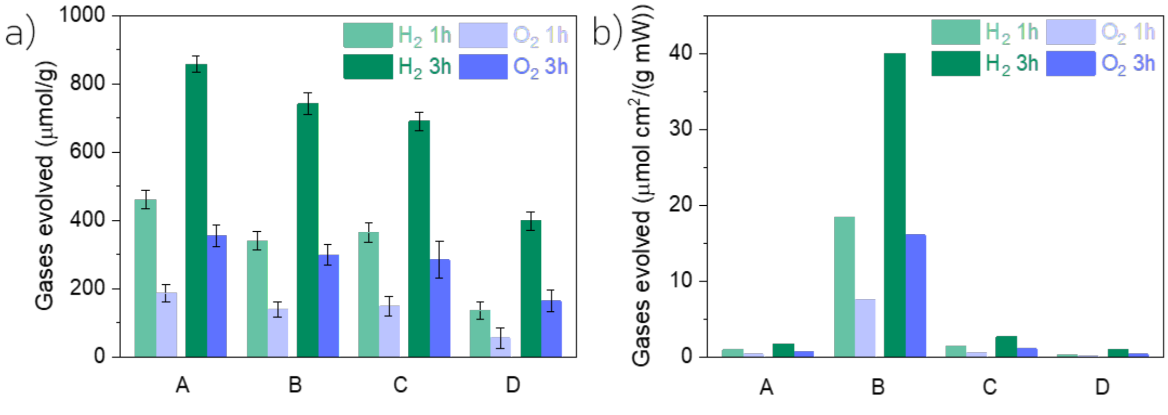

Figure S16. Influence of the irradiation conditions on the photocatalytic activity of UiO-66(Zr)-NH_2@MIL-88B(Fe) for OWS measured at 1 and 3 h, as indicated (a) and normalizing H_2 and O_2 production with light intensity (b). Conditions: Irradiations performed using UV-Vis (A), UV (B), simulated sunlight irradiation (filter AM 1.5) or visible light (> 400 nm) (C) and visible irradiation (D). Reaction conditions: photocatalyst (10 mg), H_2O (20 mL), and 35 °C.

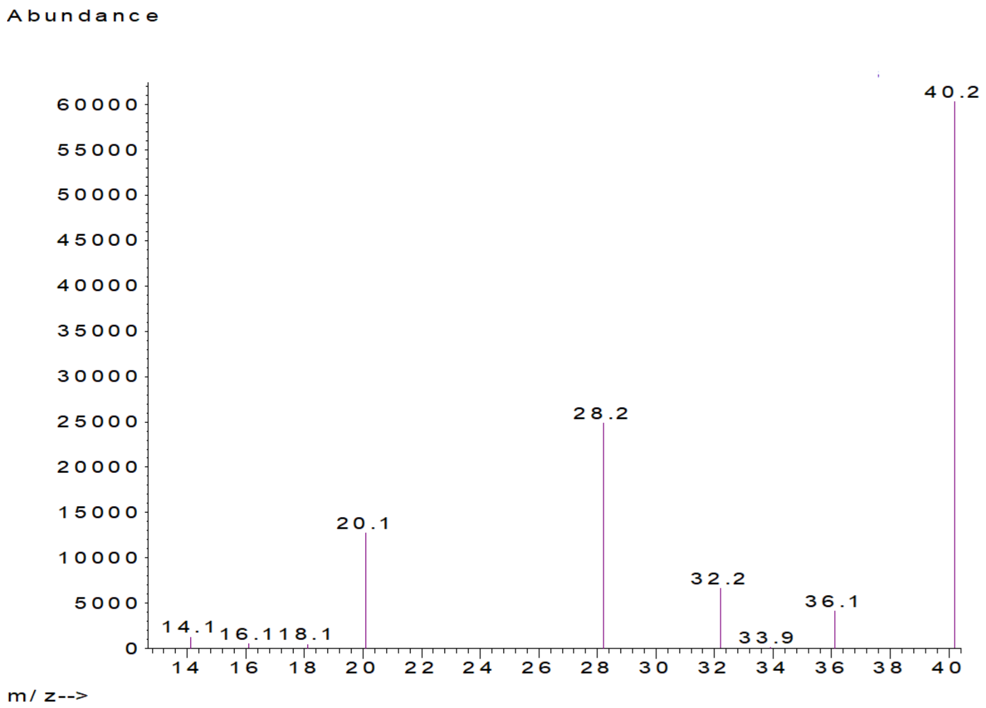

Figure S17. Mass spectrum obtained after the overall splitting reaction using labelled $H_2^{18}O$ and UiO-66(Zr)-NH_2@MIL-88B(Fe) as photocatalyst. Reaction conditions: photocatalyst (2 mg), $H_2^{18}O$ (1 mL) and simulated sunlight irradiation (150 W Hg-Xe lamp and AM 1.5G filter) during 22 h. Note: m/z 36 corresponds to $^{18}O_2$, the presence of air in the syringe during the injection is observed due to the signal at m/z 32 and 28, respectively corresponding to $^{16}O_2$ and $^{14}N_2$, as well as the peak at m/z 40 corresponding to the argon present in the reactor. Other signals at m/z 20 and as m/z 18 indicates the presence of vapours of $H_2^{18}O$ and single ^{18}O atoms, respectively, single ^{16}O atom at m/z 16 and single nitrogen atoms at m/z 14.

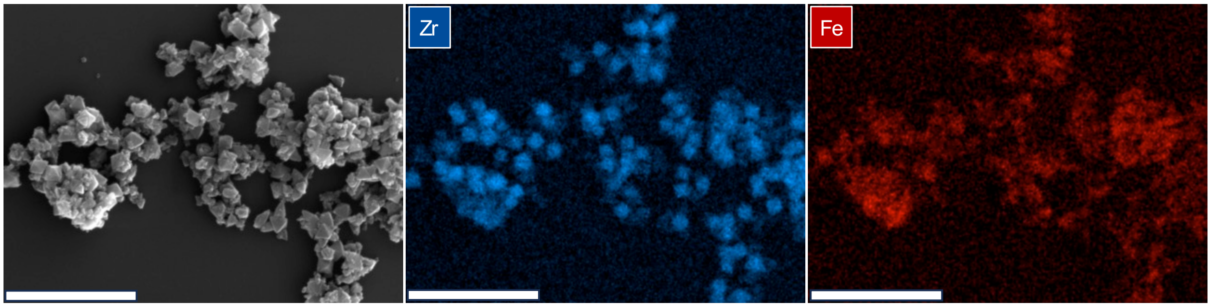

Figure S18. SEM images and elemental mapping of UiO-66(Zr)-NH_2@MIL-88B(Fe) after 5 cycles of OWS. Scale bar 2.5 μm.

Table S3. Metal leaching as determined by ICP-OES analysis of the remaining solutions after exposing UiO-66(Zr)-NH_2@MIL-88B(Fe) to photocatalytic OWS and HER conditions.

	Iron leaching (wt%)	Zirconium leaching (wt%)
OWS (water, 35 ºC, 3h)	0.05	negligible
HER (water:methanol 8:2, 35 ºC, 3h)	0.001	negligible

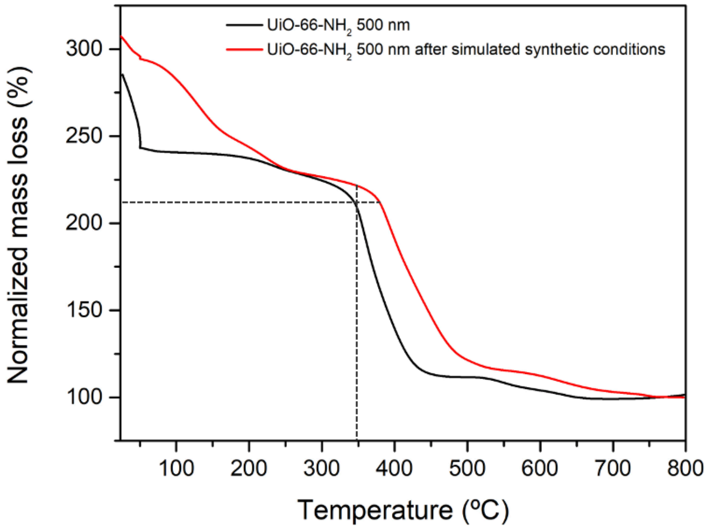

Figure S19. TGA profiles for former UiO-66(Zr)-NH_2 and the same NPs after exposure to epitaxial conditions (dispersed in DMF and heated to 100 °C for 12h).

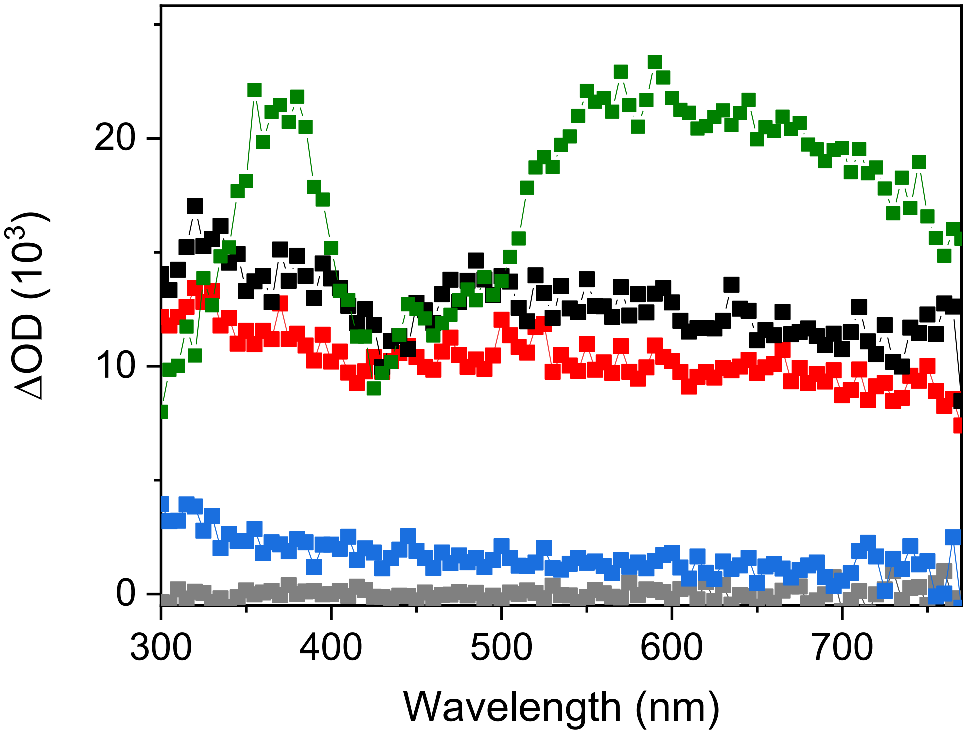

Figure S20. Transient absorption spectra of UiO-66(Zr)-NH_2@MIL-88B(Fe) upon laser excitation at 266 nm recorded at 30 ns (green), 70 ns (black), 90 ns (red), 500 ns (blue) and 3 µs (grey).

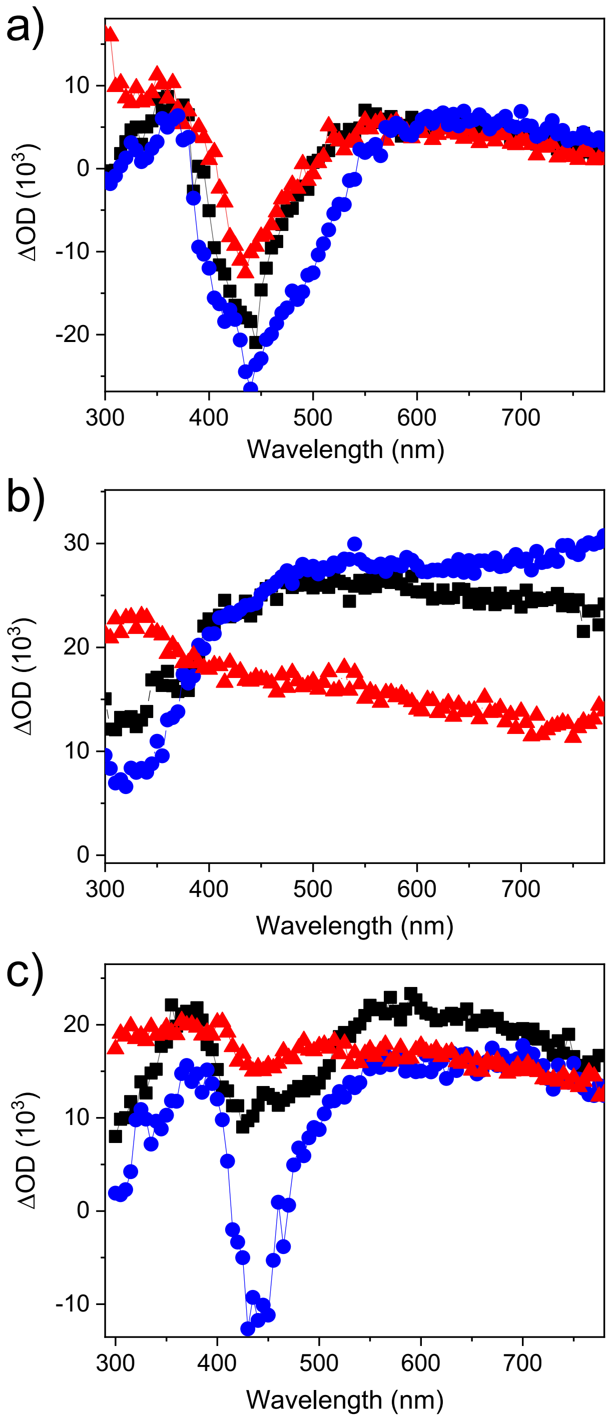

Figure S21. Transient absorption spectra of suspensions of a) UiO-66(Zr)-NH_2, b) MIL-88B(Fe) and c) UiO-66(Zr)-NH_2@MIL-88B(Fe) in acetonitrile recorded 30 ns after 266 nm laser

excitation in the presence of argon (black squares), methanol (blue spheres) or oxygen (red triangles).

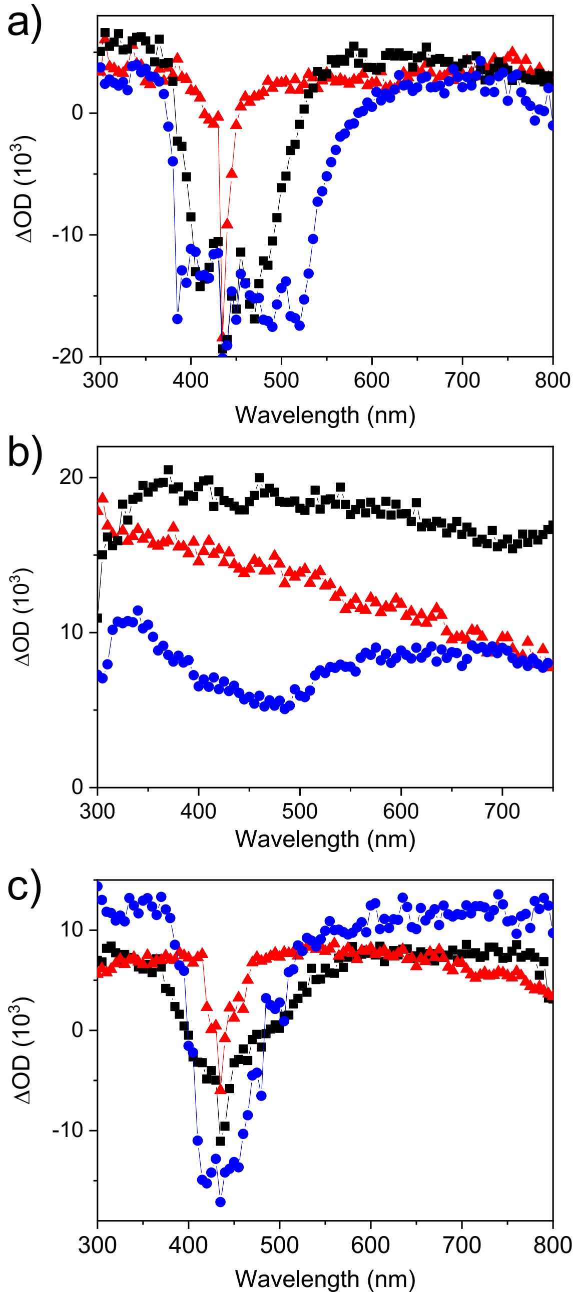

Figure S22. Transient absorption spectra of suspensions of a) UiO-66(Zr)-NH_2, b) MIL-88B(Fe) and c) UiO-66(Zr)-NH_2@MIL-88B(Fe) in acetonitrile recorded 0.036 μs after 355 nm laser excitation in the presence of argon (black squares), methanol (blue spheres) or oxygen (red triangles).

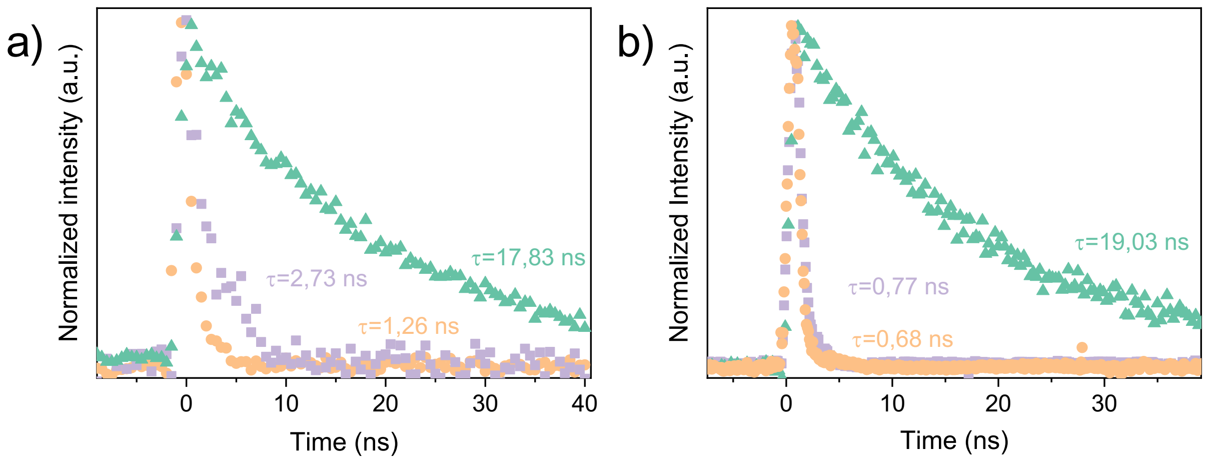

Figure S23. TRPL spectra obtained for UiO-66(Zr)-NH_2 (purple squares), MIL-88B(Fe) (orange spheres) and UiO-66(Zr)-NH_2@MIL-88B(Fe) (green triangles) after excitation at 340 nm (a) or 266 nm (b). The numbers in the graphs indicate the estimate PL lifetimes from the corresponding decay traces.

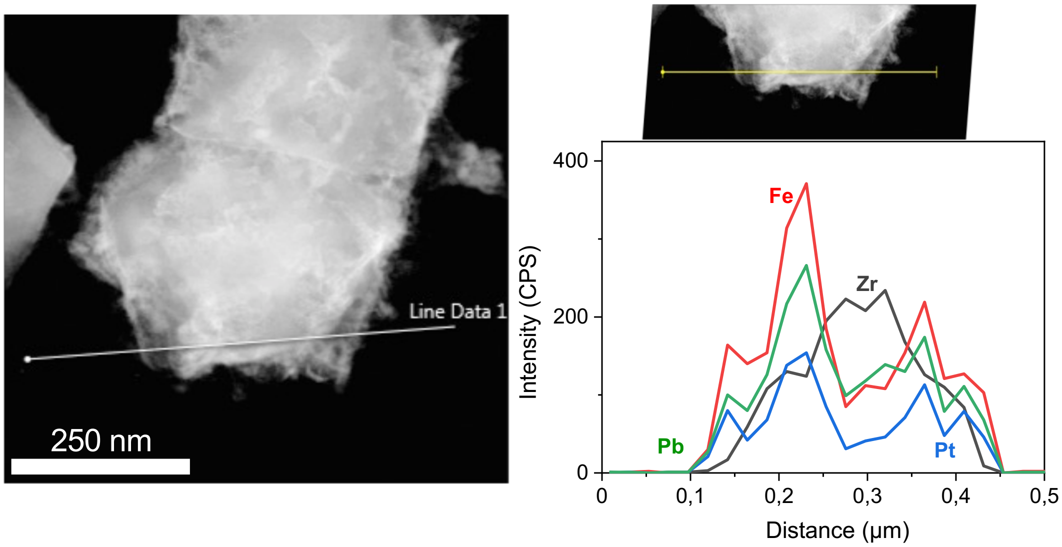

Figure S24. STEM image and line scan EDX analysis for UiO-66(Zr)-NH_2@MIL-88B(Fe) supported Pt and PbOx NPs along the path highlighted by the yellow line. The distribution of Fe, Zr, Pb and Pt is displayed on the graph.

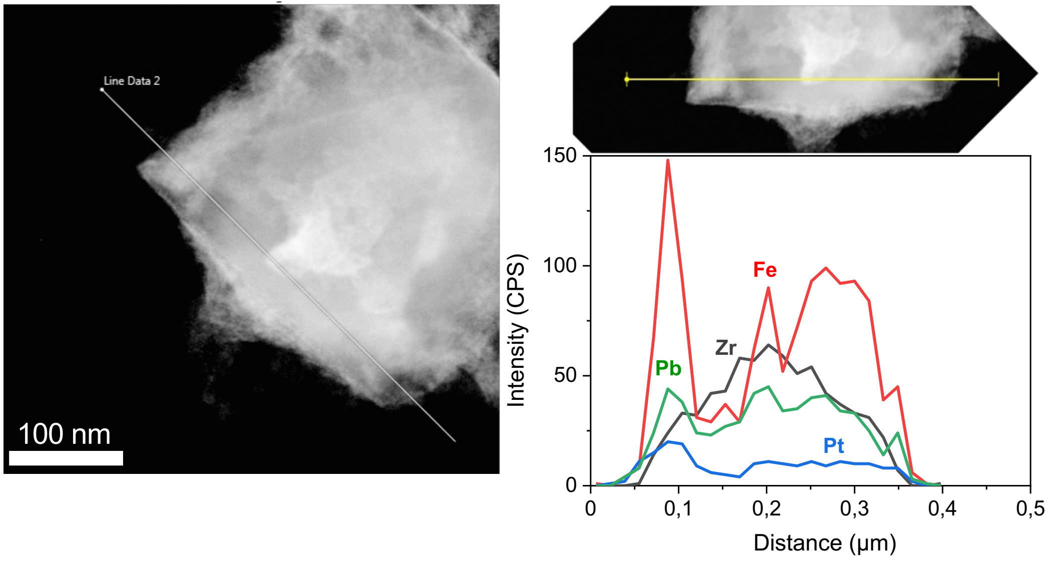

Figure S25. STEM image and line scan EDX analysis for UiO-66(Zr)-NH_2@MIL-88B(Fe) supported Pt and PbOx NPs along the path highlighted by the yellow line. The distribution of Fe, Zr, Pb and Pt is displayed on the graph.

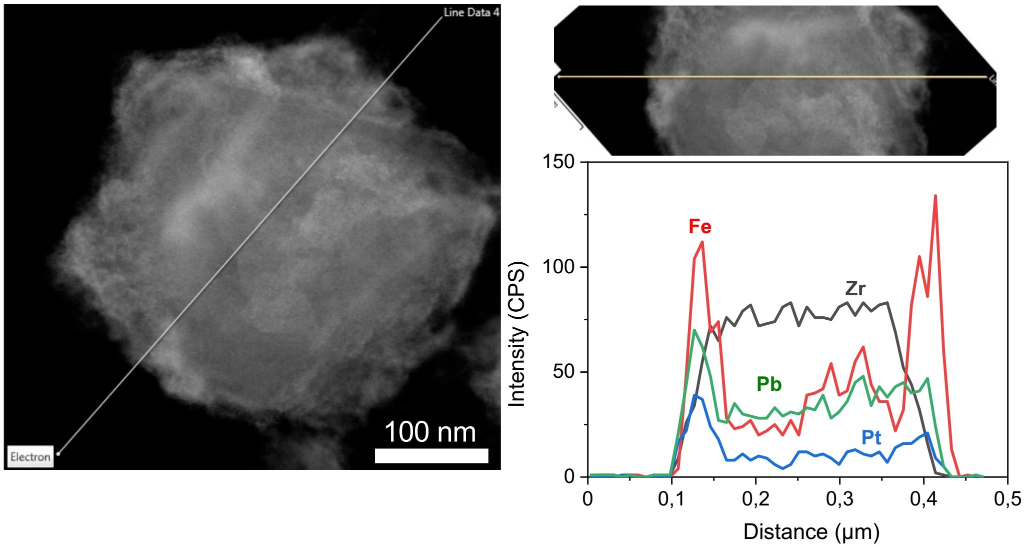

Figure S26. STEM image and line scan EDX analysis for UiO-66(Zr)-NH_2@MIL-88B(Fe) supported Pt and PbOx NPs along the path highlighted by the yellow line. The distribution of Fe, Zr, Pb and Pt is presented on the graph.

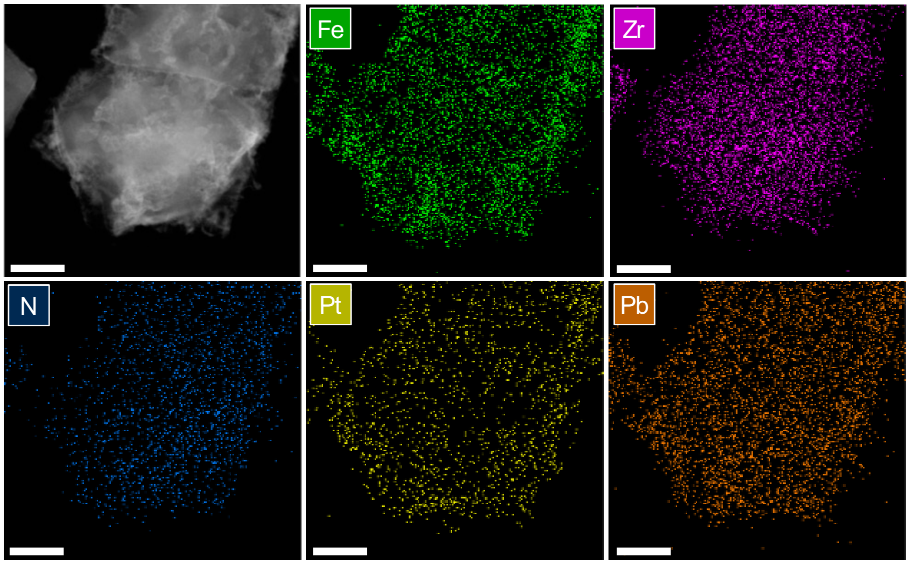

Figure S27. STEM image and elemental EDX mapping for UiO-66(Zr)-NH_2@MIL-88B(Fe) supported Pt and PbOx. Scale bar 100 nm.

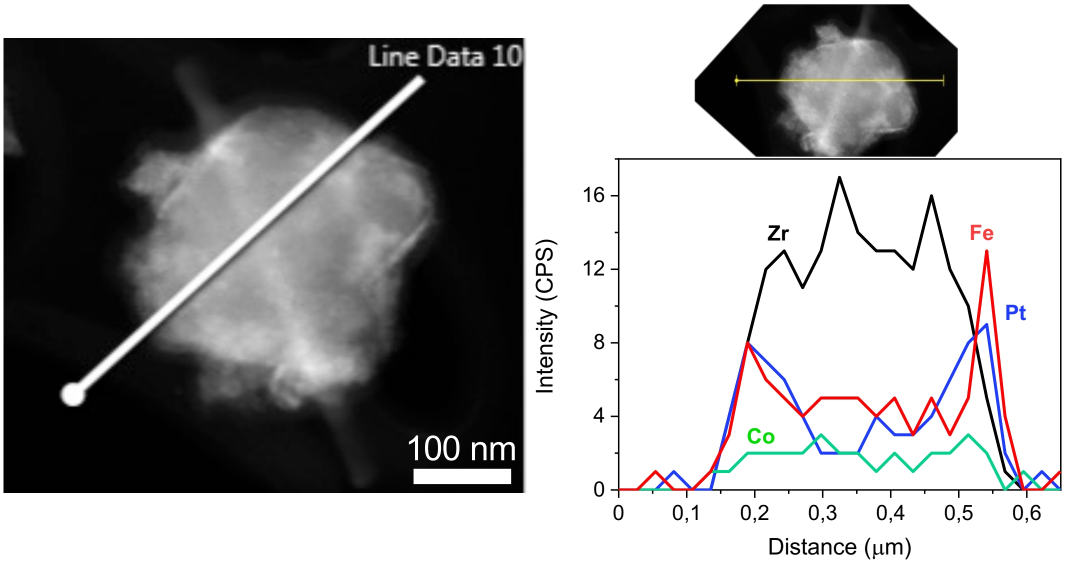

Figure S28. STEM image and line scan EDX analysis for UiO-66(Zr)-NH_2@MIL-88B(Fe) supported Pt and CoOx NPs along the path highlighted by the yellow line. The distribution of Fe, Co, Pb and Pt is presented on the graph.

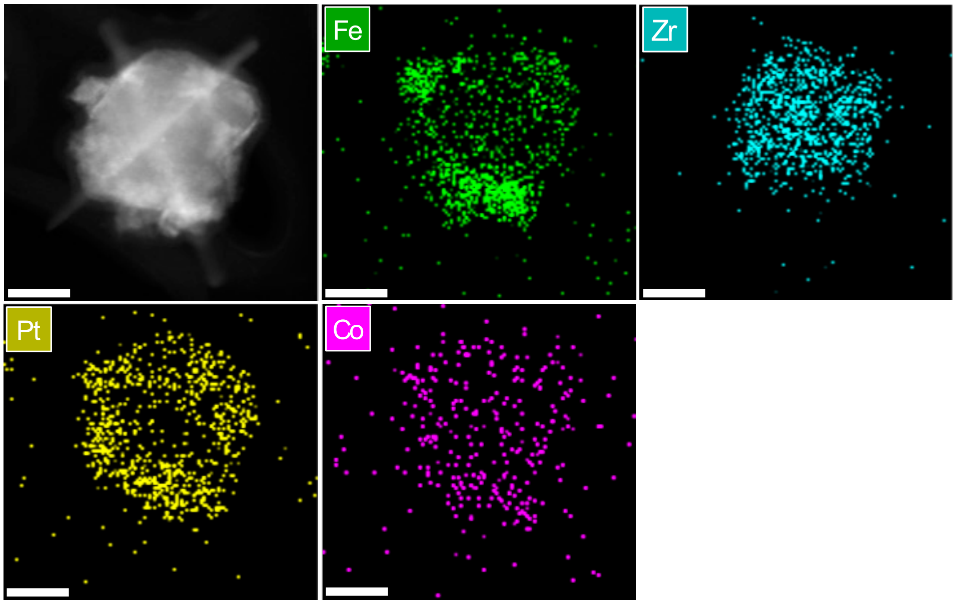

Figure S29. STEM image and elemental EDX mapping for UiO-66(Zr)-NH_2@MIL-88B(Fe) supported Pt and CoOx NPs. Scale bar 100 nm.

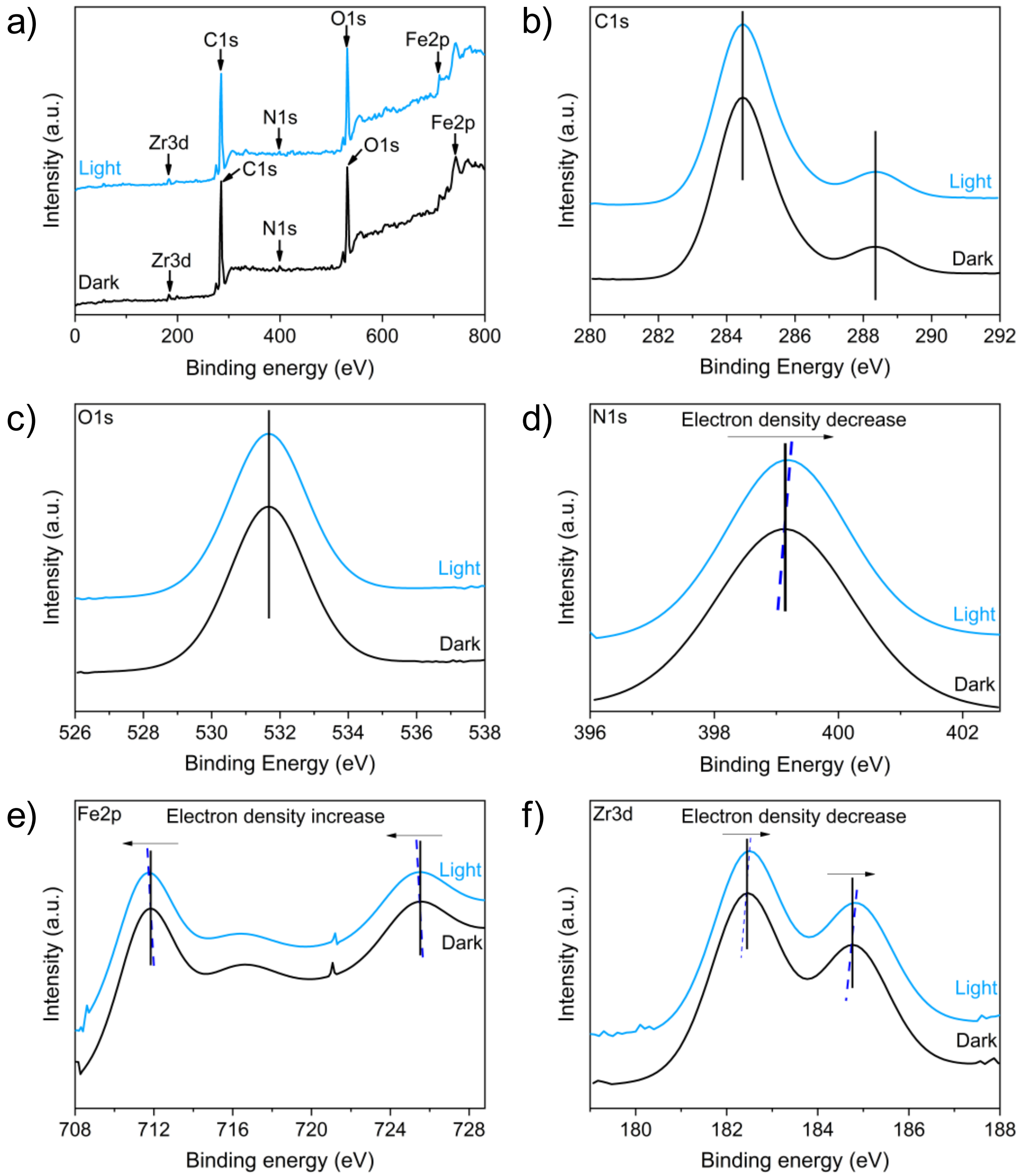

Figure S30. XPS survey (a) and *in situ* high resolution XPS signals of C 1s (b), O 1s (c), N 1s(d), Fe 2p (e) and Zr 3d (f) recorded before (black line) and after irradiation (light blue line) for UiO-66(Zr)-NH_2@MIL-88B(Fe).

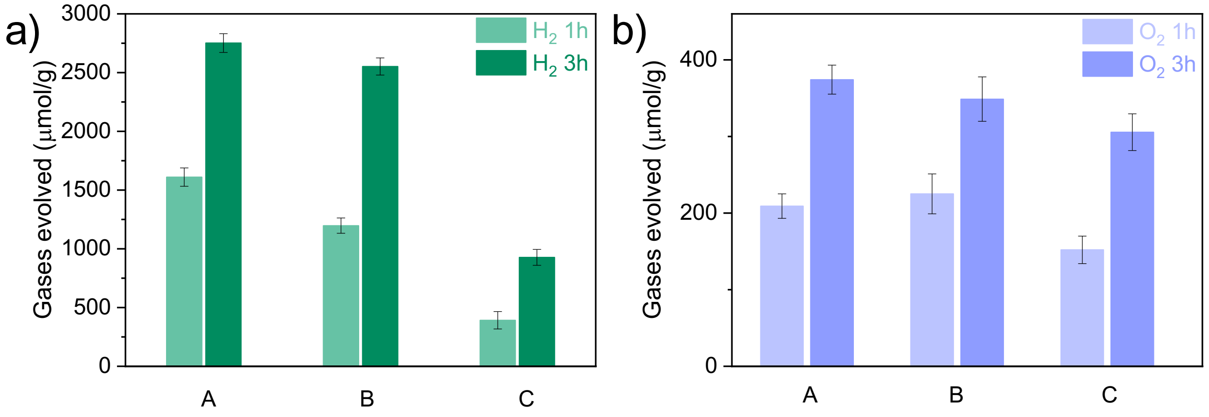

Figure S31. a) Photocatalytic HER at 1h and 3h of reaction time employing UiO-66(Zr)-NH_2@MIL-88B(Fe) and different quantities of MeOH A) 2 mL, B) 0.6 mL and C) 0.2 mL. Reaction conditions: photocatalyst (5 mg), H_2O (8 mL) and 25 °C. b) Photocatalytic OER at 1h and 3h of reaction time employing A) 700 mg, B) 300 mg and C) 50 mg of $Na_2S_2O_8$. Reaction conditions: photocatalyst (10 mg), H_2O (20 mL), and 25 °C.

■ REFERENCES

(1) Zhao, Y.; Zhang, Q.; Li, Y.; Zhang, R.; Lu, G. Large-Scale Synthesis of Monodisperse UiO-66 Crystals with Tunable Sizes and Missing Linker Defects via Acid/Base Co-Modulation. *ACS Appl. Mater. Interfaces* **2017**, *9* (17), 15079–15085. https://doi.org/10.1021/acsami.7b02887.

(2) Kwon, O.; Kim, J. Y.; Park, S.; Lee, J. H.; Ha, J.; Park, H.; Moon, H. R.; Kim, J. Computer-Aided Discovery of Connected Metal-Organic Frameworks. *Nat. Commun.* **2019**, *10*, 1–8. https://doi.org/10.1038/s41467-019-11629-4.

(3) Lázaro, I. A. A Comprehensive Thermogravimetric Analysis Multifaceted Method for the Exact Determination of the Composition of Multifunctional Metal-Organic Framework Materials. *Eur. J. Inorg. Chem.* **2020**, *2020* (45), 4284–4294. https://doi.org/10.1002/ejic.202000656.
